# Abundance and functional diversity of riboswitches in microbial communities

**DOI:** 10.1186/1471-2164-8-347

**Published:** 2007-10-01

**Authors:** Marat D Kazanov, Alexey G Vitreschak, Mikhail S Gelfand

**Affiliations:** 1 Institute for Information Transmission Problems (the Kharkevich Institute) RAS, Bolshoi Karetnyi per. 19, Moscow, 127994, Russia; 2 Faculty of Bioengineering and Bioinformatics, Moscow State University, Moscow 119992, Russia

## Abstract

**Background:**

Several recently completed large-scale enviromental sequencing projects produced a large amount of genetic information about microbial communities ('metagenomes') which is not biased towards cultured organisms. It is a good source for estimation of the abundance of genes and regulatory structures in both known and unknown members of microbial communities. In this study we consider the distribution of RNA regulatory structures, riboswitches, in the Sargasso Sea, Minnesota Soil and Whale Falls metagenomes.

**Results:**

Over three hundred riboswitches were found in about 2 Gbp metagenome DNA sequences. The abundabce of riboswitches in metagenomes was highest for the TPP, B_12 _and GCVT riboswitches; the S-box, RFN, YKKC/YXKD, YYBP/YKOY regulatory elements showed lower but significant abundance, while the LYS, G-box, GLMS and YKOK riboswitches were rare. Regions downstream of identified riboswitches were scanned for open reading frames. Comparative analysis of identified ORFs revealed new riboswitch-regulated functions for several classes of riboswitches. In particular, we have observed phosphoserine aminotransferase *serC *(COG1932) and malate synthase *glcB *(COG2225) to be regulated by the glycine (GCVT) riboswitch; fatty acid desaturase *ole1 *(COG1398), by the cobalamin (B_12_) riboswitch; 5-methylthioribose-1-phosphate isomerase *ykrS *(COG0182), by the SAM-riboswitch. We also identified conserved riboswitches upstream of genes of unknown function: thiamine (TPP), cobalamine (B_12_), and glycine (GCVT, upstream of genes from COG4198).

**Conclusion:**

This study demonstrates applicability of bioinformatics to the analysis of RNA regulatory structures in metagenomes.

## Background

Recent advances in sequencing technologies have led to a significant progress in studies of organisms in their natural habitats [1,2]. While a vast majority of currently sequenced prokaryotic organisms are culturable, they constitute less than 1% of all microbial species [3]. New sequencing methods allow one to extract and clone DNA directly from environmental samples. It makes it possible to sequence uncultured microbes, obligate parasites and symbionts. Genomic libraries created by new methods may contain DNA from many different species. This opens a new direction in sequencing, called metagenomics, which provides an opportunity for studies of microbial communities with applications in ecology, biotechnology and medicine. To date, several large-scale environmental sequencing projects have been completed. The first large project was the metagenomic sequencing project of the surface-water microbial community of the Sargasso Sea [4]. This microbial community was found to be extremely complex and diversified: the analysis of 1.6 Gbp of DNA sequence revealed over 1.2 millions genes from more than 1800 species, including 148 new species. Two other metagenomic projects [5], completed in early 2005, considered microbial communities from the surface soil of a Minnesota farm and from whale skeletons found at 500 m water depth in two different oceans. While the surface water of Sargasso Sea represents a nutrient-poor environment, agricultural soil and deep-sea whale skeletons, also known as 'whale falls', are nutrient-rich environments. The two latter projects together produced about 300 Mbp DNA sequence from more than 3000 genomes. Thus, all datasets from these projects, referred to as 'metagenomes', represent genetic information about microbial communities from different environments, including numerous known and unknown species.

In this study we consider the distribution of riboswitches in microbial communities. The riboswitches are highly conserved RNA structures that regulate gene expression without involvement of protein factors [6-9]. The riboswitch structure can be divided logically and structurally into sensory and regulatory domains. The sensory domain is a natural aptamer that selectively recognizes a target metabolite and thus indirectly estimates its concentration. After binding of an effector molecule, the sensory domain undergoes structural changes that cause simultaneous restructuring of the regulatory domain. In most cases these changes lead to repression of gene expression by transcription termination or inhibition of translation initiation. The known riboswitches are involved in regulation of numerous fundamental metabolic pathways [6,10] and have been found not only in bacterial genomes, but also in archaeal [11] and eukaryotic [12] genomes. In addition to transcription and translation, riboswitches regulate splicing [13] and RNA cleavage [14]. All that suggests that riboswitches represent one of the oldest regulatory systems [6]. On the other hand, recently discovered new classes of riboswitches [15,16] with a narrow taxonomic distribution likely have emerged a relatively short time ago.

As mentioned above, sequencing of environmental samples yields DNA fragments from both known and unknown members of microbial communities. Thus, metagenomes are an appropriate source for estimation of the riboswitch abundance in microbial communities and the diversity of their functions. The average length of DNA fragments from the three analyzed metagenomes is about 1000 bp. The riboswitch length (≈100–200 bp) and conservation level are sufficient for their reliable identification. On the other hand, in most cases the DNA fragments include both a riboswitch and a considerable part of the regulated gene. Here we present the analysis of most known types of riboswitches in DNA sequences from three large-scale environmental sequencing projects (further referred to as Sargasso Sea, Minnesota Soil and Whale Falls). We annotated functions of genes located downstream of identified riboswitches by comparative analysis. We also predicted the taxonomy origin of these DNA fragments and thus estimated the abundance of riboswitches in various taxonomic groups.

## Results and discussion

Scanning of metagenomes with patterns describing eleven riboswitch classes resulted in 311 candidate riboswitches (the corresponded patterns and alignments of identified riboswitches are presented in Additional files 9, 10, 11, 12, 13, 14, 15, 16, 17, 18, 19). The fraction of riboswitch-regulated genes in all three metagenomes was 0.03 respectively. The distribution of candidate riboswitches and riboswitch classes identified in the three metagenomes is shown in Table 1.

**Table 1:** The distribution of identified riboswitches classes over three metagenomes and the total number of riboswitches in each class


Riboswitch class	Sargasso Sea		Minnesota Soil		Whale Falls		Total	

S-box	3^a)^	2.5^b)^	5^a)^	28^b)^	4^a)^	33^b)^	12^a)^	21^c)^
RFN	2	1.6	2	11	3	25	7	12.5
LYS	1	0.8	-	-	-	-	1	0.26
B_12_	32	27	14	78	15	125	61	77
THI-box	60	50	14	78	17	142	91	90
G-box	-	-	1	0.8	-	-	1	0.26
GLMS	-	-	1	6	1	8	2	5
GCVT	106	89	4	22	9	75	119	62
YKKC/YXKD	-	-	1	6	4	33	5	13
YKOK	-	-	1	6	-	-	1	2
YYBP/YKOY	2	1.7	6	33	3	25	11	20

Total	206	1.7	49	2.7	56	4.7	311	3

^a) ^Number of identified riboswitches.

^b) ^The ratio of the number of identified riboswitches to the number of genes in a metagenome by the factor of ×10^-6 ^(except for the 'Total' row, where the factor is ×10^-4^).

^c) ^The average fraction of riboswitch-regulated genes in three metagenomes by the factor of ×10^-6 ^(except for the 'Total' row, where the factor is ×10^-4^).

The distribution of the riboswitch classes was as follows: the thiamine (THI-element), cobalamin (B_12_-element) and glycine (GCVT) riboswitches were the most abundant; the methionine (S-box), riboflavin (RFN-element), YKKC/YXKD and YYBP/YKOY riboswitches were less abundant; and only a few cases of the lysine (L-box or LYS-element), purine, GLMS and YKOK riboswitches were observed. Table 2 shows the distribution of identified riboswitches in taxonomic groups. This table also includes the values that were normalized by the total size of fragments from the taxonomic groups. The normalized frequencies of riboswitch occurrence are expressed as the number of riboswitches per 10^5 ^bp. One can see that riboswitches are abundant in the *α*-,*β*-,*γ*-Protebacteria, Firmicutes and Bacteroidetes/Chlorobi. In other taxonomic groups present in the three considered metagenomes the riboswitches are rare. The distribution of particular riboswitch types is not uniform: the THI-element is relatively more abundant in *γ*-Proteobacteria and Firmicutes, the B_12_-box, in *β*/*γ*-Proteobacteria and Bacteroidetes/Chlorobi, and the glycine riboswitch, in *α*/*β*-Proteobacteria and Firmicutes. These observations are described below in more detail.

**Table 2:** The distribution of identified riboswitches over taxonomic groups and riboswitch classes

Riboswitch Class	*α*		*β*		*γ*		*δ*		B/C		Cya		Frm		Act		Unk	
S-box	-	-	-	-	-	-	-	-	4	1.5	2	0.8	-	-	1	0.8	5	0.13
RFN	2	0.1	-	-	1	0.1	1	0.4	-	-	-	-	-	-	-	-	3	0.1
LYS	-	-	-	-	1	0.1	-	-	-	-	-	-	-	-	-	-	-	-
B_12_	12	0.6	3	1.7	13	1.4	1	0.4	5	1.9	-	-	-	-	-	-	27	0.7
THI-box	17	0.9	2	1.1	32	3.5	-	-	3	1.1	1	0.4	3	2.0	-	-	33	0.9
G-box	-	-	-	-	-	-	-	-	-	-	-	-	-	-	-	-	1	0.03
GLMS	-	-	-	-	-	-	-	-	-	-	-	-	-	-	-	-	2	0.05
GCVT	88	4.7	4	2.2	8	0.9	-	-	-	-	-	-	5	3.3	-	-	14	0.4
YKKC/YXKD	1	0.05	1	0.6	1	0.1	2	0.8	-	-	-	-	-	-	-	-	-	-
YKOK	-	-	-	-	-	-	-	-	1	0.4	-	-	-	-	-	-	-	-
YYBP/YKOY	1	0.05	1	0.6	2	0.2	-	-	-	-	-	-	-	-	-	-	7	0.2

Total	121	6.4	11	6.1	58	6.3	4	1.6	13	4.8	3	1.2	8	5.3	1	0.8	92	2.4

The first subcolumn in each column is the number of riboswitches, the second subcolumn is the normalized frequences of riboswitch occurrence (the number of riboswitches per 10^5 ^bp). Columns: *α*,*β*,*γ*,*δ *– classes of Proteobacteria, B/C – Bacteroidetes/Chlorobi, Cya – Cyanobacteria, Frm – Firmicutes, Act – Actinobacteria, Unk – unknown taxonomy.

### S-box (SAM-riboswitch)

The S-adenosylmethionine-dependent riboswitches [17-21] identified in the analyzed metagenomes are presented in Additional file 1. All annotated proteins possibly regulated by S-boxes, i.e. located downstream of the identified riboswitches, except one (COG0182) were observed in *B. subtilis *and *Escherichia coli *earlier [21]. One protein remains unannotated because the search against the Genbank database retrieved no result. The taxonomy of seven out of twelve riboswitch-containing DNA fragments was successfully predicted. The methionine riboswitch occurs not only in Firmicutes and Actinobacteria groups, as previously thought [21], but also in the Bacteroidetes/Chlorobi and Cyanobacteria groups. Only one out of seven classified DNA fragments was assigned to the Actinobacteria group, while other were assigned to the Bacteroidetes/Chlorobi and Cyanobacteria groups despite the fact that the estimated total size of the Firmicutes and Actinobacteria groups in the Sargasso Sea metagenome is approximately equal to the estimated total size of the Bacteroidetes/Chlorobi and Cyanobacteria groups [4].

One protein was significantly similar to the predicted translation initiation factor 2B subunit from the eIF-2B *α*/*β*/*δ *family (COG0182). However, recently the YkrS protein from *B. subtilis *belonging to this family was characterized as a 5-methylthioribose-1-phosphate isomerase [22]. This enzyme participates in the methionine salvage pathway, and this is consistent with its regulation by S-boxes.

### RFN-element (FMN-riboswitch)

The distribution of FMN-dependent riboswitches (RFN-element) [23-26] (Additional file 2) is consistent with results obtained for complete bacterial genomes [26]. In two out of seven cases, FMN-riboswitches were identified upstream of a single gene *ribB *not belonging to a riboflavin biosynthesis operon. Based on the gene taxonomic affinity these riboswitches belong to the Proteobacteria. Three times, an FMN riboswitch was identified upstream of the gene *ribH*, and it could not be determined whether this gene belongs to a riboflavin biosynthesis operon because of the small fragment size. These DNA fragments, except one unclassified case, were assigned to the *α*-Proteobacteria. Two remaining FMN riboswitches were observed upstream of the genes *ribD *and *ribC*. These genes may be the first genes of riboflavin operons [26], but again, whether this is the case could not be determined because of insufficient size of DNA fragments.

### B_12_-element

Cobalamin riboswitches (B_12_) [27-31] observed in three metagenomes are presented in Additional file 3. The genes preceded by identified B_12_-elements could be divided by function into three groups. The first group contains cobalamin biosynthesis genes *cobW*, *hupE*, *cobU*. The second group, which is the most numerous one, are cobalt and cobalamin transporters *btuB*, *btuC*, *fecB*. The third group if formed by *metE*, a B_12_-independent isozyme of a B_12_-dependent enzyme. It was shown [31] that if both isozymes are encoded in one genome, then the expression of the B_12_-independent isozyme is repressed in the presence of cobalamin. The fatty-acid desaturase gene *ole1 *that was for the first time observed to be regulated by a cobalamin riboswitch, may also belong to such a pair of isozymes. If this hypothesis is correct, there must exist a corresponding B_12_-dependent isozyme. For 24 identified B_12_-elements the regulated gene could not be determined: in three cases because riboswitches were located at the end of DNA fragments, in six cases because of the absence of open reading frames in the downstream region, and in fifteen cases because of the absence of similar genes in the database. Two of the latter fifteen genes are significantly similar to each other.

The distribution of B_12_-elements over taxonomic groups is slightly different from that observed for complete bacterial genomes [31]. In the latter, B_12_-elements were found mainly in the Proteobacteria, Firmicutes and Actinobacteria, and the number of B_12_-elements in the Firmicutes and Actinobacteria was only slightly less than the number of B_12_-elements in the Proteobacteria. Although the percentage of the Firmicutes and Actinobacteria groups is not negligible (about 10% in the Sargasso Sea metagenome), none of 32 cobalamin riboswitch-containing DNA fragments belonged to the Firmicutes or Actinobacteria.

### THI-element (TPP-riboswitch)

Thiamine pyrophosphate (TPP)-dependent riboswitches [11-13,32,33] identified in three metagenomes are presented in Additional file 4. Among the thiamine biosynthesis genes, most TPP-riboswitches were found upstream of the *thiC *and *thiM *genes. These genes are known to occur as first genes of THI-element-regulated thiamine biosynthesis operons [11], but we were able to confirm this only in one case because of the small size of DNA fragments. One THI-element was found upstream of another thiamine biosynthesis gene, *thiD*. This gene did not seem to be a part of an operon.

Another group of TPP-riboswitch-regulated genes are thiamine transporters. Most TPP-riboswitches were found upstream of the *thiB *gene. We were able to examine whether these genes belong to operons, as found earlier [11], in only three cases, where it was a part of the *thiBP *operon. This agrees with the analysis of complete bacterial genomes [11]. Other identified transporters regulated by THI-elements were *omr *(homolog of the outer membrane receptor *btuB*), ABC-type transporter *thiXYZ *from the nitrate/sulfonate/bicarbonate transporter family, and *thiV*, homologous to the Na+/panthothenate symporter gene *panF *[11].

Functions of 23 other TPP-riboswitches were not determined: in one case because of the absence of open reading frames in the downstream region, in ten cases because riboswitches were located at the end of DNA fragments, and in twelve cases because of the absence of similar genes in the databases. Two of the latter twelve genes were significantly similar to each other.

### GCVT

The glycine riboswitch [34,35] is highly abundant and, as expected [34,35], was mainly identified in upstream regions of genes encoding the glycine cleavage system (Additional file 5). In 70 out of 119 cases (≈59%) the GCVT riboswitch occurred upstream of the *gcvT *gene. In 25 out of these 70 cases this gene was a part of the operon *gcvTHP*, whereas for other DNA fragments the operon structure was not discernible because of their small size. The second frequently observed (≈20%) glycine-riboswitch regulated gene was the malate synthase gene *glcB *involved in the pyruvate metabolism. This study provides the first such example. Most of these genes belonged to DNA fragments from the Sargasso Sea metagenome, moreover, 78 out of 94 (≈83%) DNA fragments apparently belonged to one specie *Candidatus Pelagibacter ubique *from the *α*-Proteobacteria. Other annotated genes form ≈7% of all identified genes downstream of glycine riboswitches. All of them, except *serC*, already have been observed under glycine riboswitch regulation [34] and are involved in the glycine or pyruvate metabolism. All these glycine riboswitches had tandem aptamer domains, as in [35]. All DNA fragments containing these riboswitches (with one exception) presumably belong to the Proteobacteria.

For remaining ≈14% of the identified glycine riboswitches, the regulated functions could not be identified. The search against the COG database showed that five genes were significantly similar to genes from the COG4198 cluster, described as a group of uncharacterized conserved proteins. Interestingly, the structure of riboswitches for these seven cases had only one aptamer domain. The DNA fragments of these proteins presumably belong to the Firmicutes. In twelve remaining cases protein sequences could not be determined because riboswitches were located at the end of DNA fragments.

### YKKC/YXKD

The search for the YKKC/YXKD riboswitches (Additional file 6) confirmed known regulated functions of these elements [34]. Four out of five identified riboswitches were upstream of two components of an ABC-type transport system from the nitrate/sulfonate/bicarbonate family. One YKKC/YXKD riboswitch was identified upstream of the amino acid transporter *potE*. All YKKC/YXKD riboswitches were found in DNA fragments from the Proteobacteria.

### YYBP/YKOY

The YYBP/YKOY riboswitch was less abundant than the GCVT element in metagenomes (Additional file 7) unlike the situation in complete genomes [34]. On the other hand, the regulated functions were essentially the same as in the latter [34]. All annotated genes observed downstream of the identified riboswitches could be classified into three groups: two encoding predicted membrane proteins, and one, the *terC *genes. In three cases no open reading frames were found in the downstream region. Most YYBP/YKOY riboswitch-containing DNA fragments belong to the Proteobacteria.

### L-box (LYS-element), G-box, GLMS, YKOK

The lysine (L-box) [36-40] and purine riboswitch (G-box) [41-45] as well as the GLMS [14,34] and YKOK [34] riboswitches were rare in metagenomes. The only lisine riboswitch were identified in the Sargasso Sea metagenome and presumably belongs to the *γ*-Proteobacteria. This riboswitch was located upstream of the predicted lysine transporter from the *Na+:H+ antiporter *superfamily [40]. The function of the only purine riboswitch-regulated gene was not recognized because of the absence of similar genes in the databases. Two GLMS riboswitches were observed: one upstream of the *glmS *gene, and one at the end of a DNA fragment. Of two observed YKOK riboswitches, one was upstream of a gene similar to the Mg/Co/Ni transporter *mgtE *(COG2239), and one, upstream of a gene with unknown function.

## Conclusion

The riboswitch counts in the Sargasso Sea, Minessota Soil and Whale Falls bacterial communities estimated here generally agree with the riboswitch abundance in complete bacterial genomes [11,21,26,31,34,40]. In the bacterial communities, the THI-elements, B_12_-elements and GCVT riboswitches are the most abundant. The two former riboswitches are highly abundant in complete bacterial genomes as well [11,31], whereas the GCVT riboswitch was not the most frequent one among computationally discovered riboswitches GLMS, GCVT, YKKC/YXKD, YKOK and YYBP/YKOY [34]. The YYBP/YKOY riboswitch was characterized as the most abundant one among these new riboswitches [34], however it occurs in metagenomes less frequently than the THI, B_12 _and GCVT elements. The S-boxes, RFN-elements, YYBP/YKOY and YKKC/YXKD riboswitches demonstrated lower but still significant abundance, whereas the GLMS, YKOK, lysine and purine riboswitches were rare. In general, the riboswitch frequencies weakly depend on a particular metagenome, however they are slightly higher in the Minnesota Soil and Whale Falls metagenomes then in the Sargasso Sea metagenome. The glycine riboswitch (GCVT) is an exception: its frequency in the Sargasso Sea metagenome was the highest, about fourfold higher than in the Minnesota Soil metagenome and 1.2-fold higher than in the Whale Falls metagenome. However, ≈81% of glycine riboswitches coming from the Sargasso Sea metagenome presumably belong to a single specie *Candidatus Pelagibacter ubique *from the SAR11 clade, which is known to be abundant in marine surface waters [46,47]. This example and the discrepancies between the riboswitch abundance in metagenomes and complete genomes demonstrate the influence of species frequencies in the communities on the gene and riboswitch contents of the latter.

The riboswitch-regulated functions in metagenomes in most cases coincide with those observed in complete bacterial genomes [11,21,26,31,34,40]. However, several new regulated functions were recognized for some riboswitch classes. The new functions regulated by the glycine riboswitch, phosphoserine aminotransferase (COG1932) and malate synthase (COG2225), are involved in the glycine and pyruvate metabolism, respectively. Fatty-acid desaturase (COG1398) was recognized as a new regulated function of the cobalamin riboswitch (B_12_-element). We suggest that this fatty-acid desaturase gene belongs to a pair of B_12_-dependend and B_12_-independed isozymes [31]. One more new riboswitch-regulated gene is under methionine (SAM) regulation and shows a significant similarity with the translation initiation factor 2B subunit from the eIF-2B *α*/*β*/*δ *family (COG0182); however, according to recent studies [22] the real function of this protein is 5-methylthioribose-1-phosphate isomerase.

Sometimes different riboswitches were found upstream of homologous genes. For example, components of ABC-type nitrate/sulfonate/bicarbonate transport systems homologous to *tauA *and *tauC *were found downstream of thiamine and YKKC/YXKD riboswitches. One other example is provided by *btuB *homologs from the outer membrane receptor proteins family regulated by thiamine and B_12 _riboswitches.

In addition to genes with known (or reliably predicted) functions, this study revealed several hypothetical riboswitch-regulated genes. Leaving aside relatively less reliable "orphans", that is, open reading frames preceded by riboswitches, but having no homologs, we have observed several groups of homologous genes preceded by riboswitches. The examples are two pairs of homologous proteins regulated by B_12 _and thiamine riboswitches, respectively, and five uncharacterized conserved proteins regulated by the GCVT riboswitch and belonging to COG4198 cluster.

When this study had been completed, a much larger metagenomic collectioin was published [48], and several new riboswitches were identified [[49], D. Rodionov personal communication]. This study demonstrates that metagenomics and bioinformatics can be applied to the analysis not only of genes, proteins, and metabolic pathways [50-52], but regulatory structures in natural environments not biased towards cultured organisms. We expect that the new datasets may contain not only new examples of functions regulated by known riboswiches, but new types of riboswitches as well.

## Methods

The RNA-PATTERN program [53] was used to search for RNA regulatory elements in all three metagenomes. The input RNA pattern described the RNA secondary structure and the sequence consensus motifs as a set of the following parameters: the number of helices, the length of each helix, the loop lengths and the topology of helix pairs. The appropriate patterns were created for the analyzed riboswitches and used in search procedure, see Additional files 9, 10, 11, 12, 13, 14, 15, 16, 17, 18, 19.

For the automated analysis we developed a specialized software based on the Relational Database Management System (RDBMS) Oracle 10g Express Edition [54]. It was used to load the metagenomic data, execute the RNA-PATTERN program, and support the functional annotation of regulated genes and taxonomic annotation of riboswitch-containing DNA fragments. The data processing flow is shown schematically in Additional file 8. At the first step, metagenome DNA sequence files were loaded into the database from GenBank [55]. We excluded the metagenome data belonging to the first of the seven Sargasso sea samples, because it seems to be contaminated by *Shewanella *and *Burkholderia *species [48,56]. Then, automated search for all riboswitch classes in each file was performed by invoking the RNA-PATTERN program. Search results were loaded into the database and could be viewed in an ad-hoc user interface. To simplify functional annotation of genes located downstream of identified riboswitches, the automated search of similar sequences was performed by the Entrez Programming Utilities interface [57] to the BLAST program [58]. The resulting lists of similar protein sequences were also loaded into the database and could be viewed using the same user interface. The functional annotation of proteins was performed by comparison with the COG database [59]. The organism names were extracted from each BLAST hit and the complete taxonomy of the organism was requested from the NCBI Taxonomy Browser [60]. The retrieved complete taxonomy of organisms was linked in the database to the associated BLAST hit and used to predict the taxonomy of riboswitch-containing DNA fragments. To do that, we compared the taxonomy of the several most similar hits and extracted the maximum level of the taxonomic hierarchy that was common for the considered hits. If these hits had at least one common taxonomic level of hierarchy, then we considered that taxonomy of DNA fragment was successfully predicted and assigned this taxonomic level to the DNA fragment itself.

Sequence alignments of identified riboswitches were prepared for publishing using the T_E_Xshade package [61] and an ad hoc unpublished program (MK).

## Competing interests

The author(s) declares that there are no competing interests.

## Authors' contributions

MG conceived the project. MK performed the computational analysis of the metagenome data. LV provided the program for the identification of riboswitches. MK and LV performed functional annotation. MK and MG wrote the paper. All the authors have read and approved the final manuscript.

## Supplementary Material

Additional file 1 S-boxes (SAM-riboswitches) and their regulated functions identified in three metagenomes. New functions are set in boldface. Click here for file

Additional file 2 RFN-elements (FMN-riboswitches) and their regulated functions identified in three metagenomes. Click here for file

Additional file 3 B_12_-elements and their regulated functions identified in three meta genomes. New functions are set in boldface. Click here for file

Additional file 4 Thiamine riboswitches and their regulated functions identified in three metagenomes. Click here for file

Additional file 5 Glycine riboswitches and their regulated functions identified in three metagenomes. New functions are set in boldface. Click here for file

Additional file 6 YKKC/YXKD riboswitches and their regulated functions identified in three metagenomes. Click here for file

Additional file 7 YYBP/YKOY riboswitches and their regulated functions identified in three metagenomes. Click here for file

Additional file 8 Data processing flow. Click here for file

Additional file 9 Search pattern and sequence alignment of SAM-riboswitches. Click here for file

Additional file 10 Search pattern and sequence alignment of FMN-riboswitches. Click here for file

Additional file 11 Search pattern and sequence alignment of cobalamin riboswitches. Click here for file

Additional file 12 Search pattern and sequence alignment of TPP-riboswitches. Click here for file

Additional file 13 Search pattern and sequence alignment of glycine riboswitches. Click here for file

Additional file 14 Search pattern and sequence alignment of YKKC/YXKD riboswitches. Click here for file

Additional file 15 Search pattern and sequence alignment of YYBP/YKOY riboswitches. Click here for file

Additional file 16 Search pattern and sequence alignment of lysine riboswitches. Click here for file

Additional file 17 Search pattern and sequence alignment of purine riboswitches. Click here for file

Additional file 18 Search pattern and sequence alignment of GLMS riboswitches. Click here for file

Additional file 19 Search pattern and sequence alignment of YKOK riboswitches. Click here for file
