# Supplementary material for: Abundance and functional diversity of riboswitches in microbial communities
Source: BMC Genomics. 2007 Oct 1;8:347. doi: 10.1186/1471-2164-8-347 (PMC2211319; doi:10.1186/1471-2164-8-347)
Supplement: Additional file 1 — S-boxes (SAM-riboswitches) and their regulated functions identified in three metagenomes. New functions are set in boldface. [file 1471-2164-8-347-S1.pdf]

Protein function	Gene	Number of riboswitches in metagenomes (grouped by taxonomy)					
		Sargasso Sea		Minnesota Soil		Whale Falls	
Methionine synthase I (cobalamin-dependent), methyltransferase domain (COG0646)	*metH*	Bacteroidetes/ Chlorobi	2		-	Bacteroidetes/ Chlorobi	1
Homoserine trans-succinylase (COG1897)	*metA*	Cyanobacteria	1		-		-
Homoserine dehydrogenase (COG0460)	*hom*		-	Bacteria	2		-
Homoserine acetyltransferase (COG2021)	*met2*		-		-	Bacteroidetes/ Chlorobi	1
Threonine synthase (COG0498)	*thrC*		-<br>-	Actinobacteria<br>Bacteria	1<br>1		-<br>-
O-acetylhomoserine sulfhydrylase (COG2873)	*met17*		-		-	Cyanobacteria	1
**5-methylthioribose-1-phosphate isomerase homologous to predicted translation initiation factor 2B subunit, eIF-2B alpha/beta/delta family (COG0182)**	***gcn3***		-	Bacteria	1		-
Unknown function (no similar proteins)			-		-		1

Additional file 1: S-boxes (SAM-riboswitches) and their regulated functions identified in three metagenomes. New functions are set in boldface.
