# Supplementary material for: Abundance and functional diversity of riboswitches in microbial communities
Source: BMC Genomics. 2007 Oct 1;8:347. doi: 10.1186/1471-2164-8-347 (PMC2211319; doi:10.1186/1471-2164-8-347)
Supplement: Additional file 2 — RFN-elements (FMN-riboswitches) and their regulated functions identified in three metagenomes. [file 1471-2164-8-347-S2.pdf]

Protein function	Gene	Number of riboswitches in metagenomes (grouped by taxonomy)		
		Sargasso Sea	Minnesota Sea	Whale Falls
3,4-dihydroxy-2-butanone 4-phosphate synthase (COG0108)	*ribB*	-	γ-Proteobacteria 1	δ-Proteobacteria 1
Pyrimidine reductase, riboflavin biosynthesis (COG1985)	*ribD*	Bacteria 1	-	-
Riboflavin synthase alpha chain (COG0307)	*ribC*	Bacteria 1	-	-
Riboflavin synthase beta-chain (COG0054)	*ribH*	-	Bacteria 1	α-Proteobacteria 2

Additional file 2: RFN-elements (FMN-riboswitches) and their regulated functions identified in three metagenomes.
