# Supplementary material for: Abundance and functional diversity of riboswitches in microbial communities
Source: BMC Genomics. 2007 Oct 1;8:347. doi: 10.1186/1471-2164-8-347 (PMC2211319; doi:10.1186/1471-2164-8-347)
Supplement: Additional file 3 — B12-elements and their regulated functions identified in three meta genomes. New functions are set in boldface. [file 1471-2164-8-347-S3.pdf]

| Protein function | Gene | Number of riboswitches in metagenomes (grouped by taxonomy) | | | | | |
|---|---|---|---|---|---|---|---|
| | | Sargasso Sea | | Minnesota Soil | | Whale Falls | |
| Outer membrane cobalamin receptor protein (COG4206) | *btuB* | $\alpha$-Proteobacteria | 4 | Bacteroidetes/ Chlorobi | 1 | Bacteroidetes/ Chlorobi | 3 |
| | | $\beta$-Proteobacteria | 3 | $\alpha$-Proteobacteria | 1 | $\alpha$-Proteobacteria | 1 |
| | | $\gamma$-Proteobacteria | 9 | $\gamma$-Proteobacteria | 1 | $\gamma$-Proteobacteria | 1 |
| | | | | | | $\delta$-Proteobacteria | 1 |
| Putative GTPases (G3E family) (COG0523) | *cobW* | $\alpha$-Proteobacteria | 2 | | - | | - |
| Hydrogenase/urease accessory protein (COG2370) | *hupE* | $\alpha$-Proteobacteria | 2 | | - | | - |
| ABC-type Fe3+-siderophore transport system, permease component (COG0609) | *btuC* | | - | | - | Bacteria | 1 |
| ABC-type Fe3+-hydroxamate transport system, periplasmic component (COG0614) | *fecB* | | - | Bacteria | 2 | $\alpha$-Proteobacteria | 1 |
| Adenosyl cobinamide kinase/adenosyl cobinamide phosphate guanylyltransferase (COG2087) | *cobU* | | - | | - | $\alpha$-Proteobacteria | 1 |
| | | | | | | $\gamma$-Proteobacteria | 1 |
| Methionine synthase II (cobalamin-independent) (COG0620) | *metE* | | - | | - | Bacteroidetes/ Chlorobi | 1 |
| **Fatty-acid desaturase (COG1398)** | ***ole1*** | | - | $\gamma$-Proteobacteria | 1 | | - |
| Unknown function: No ORF | | | 3 | | 3 | | - |
| Unknown function: No similar proteins | | | 6 | | 5 | | 4 |
| Unknown function: End of DNA fragment | | | 3 | | - | | - |

Additional file 3: $B_{12}$-elements and their regulated functions identified in three metagenomes. New functions are set in boldface.
