# Supplementary material for: Abundance and functional diversity of riboswitches in microbial communities
Source: BMC Genomics. 2007 Oct 1;8:347. doi: 10.1186/1471-2164-8-347 (PMC2211319; doi:10.1186/1471-2164-8-347)
Supplement: Additional file 4 — Thiamine riboswitches and their regulated functions identified in three metagenomes. [file 1471-2164-8-347-S4.pdf]

Protein function		Gene	Number of riboswitches in metagenomes (grouped by taxonomy)					
			Sargasso Sea		Minnesota Soil		Whale Falls	
ABC-type thiamine transport system, periplasmic component (COG4143)		*thiB*	α-Proteobacteria	4	γ-Proteobacteria	1	α-Proteobacteria	2
			γ-Proteobacteria	13	Bacteria	1	γ-Proteobacteria	2
			Bacteria	1				
Thiamine biosynthesis protein ThiC (COG0422)		*thiC*	γ-Proteobacteria	3	Bacteria	4	γ-Proteobacteria	4
			β-Proteobacteria	2	α-Proteobacteria	2	α-Proteobacteria	2
			Cyanobacteria	1			Bacteria	1
Outer membrane receptor proteins, mostly Fe transport (COG1629)		*btuB*	γ-Proteobacteria	9	Bacteroidetes/ Chlorobi	1	Bacteroidetes/ Chlorobi	1
			Bacteroidetes/ Chlorobi	1				
ABC-type nitrate/sulfonate/bicarbonate transport system, periplasmic component (COG0715)		*tauA*	α-Proteobacteria	2		-		-
ABC-type nitrate/sulfonate/bicarbonate transport system, ATPase component (COG1116)		*tauB*	Firmicutes	2		-		-
ABC-type nitrate/sulfonate/bicarbonate transport system, permease component (COG0600)		*tauC*	Firmicutes	1		-		-
Hydroxyethylthiazole kinase, sugar kinase family (COG2145)		*thiM*	α-Proteobacteria	2	α-Proteobacteria	1		-
Na+/proline, Na+/panthothenate symporters and related permeases (COG0591)		*panF*	Bacteria	1				
			α-Proteobacteria	1		-		-
Thiamine pyrophosphokinase (COG1564)		*thi80*		-	Bacteria	2		-
Hydroxymethylpyrimidine/phosphomethylpyrimidine kinase (COG0351)		*thiD*		-		-	α-Proteobacteria	1
Unknown function	No ORF			1		-		-
	No similar proteins			8		2		2
	End of DNA fragment			8		-		2

Additional file 4: Thiamine riboswitches and their regulated functions identified in three metagenomes.
