# Supplementary material for: Abundance and functional diversity of riboswitches in microbial communities
Source: BMC Genomics. 2007 Oct 1;8:347. doi: 10.1186/1471-2164-8-347 (PMC2211319; doi:10.1186/1471-2164-8-347)
Supplement: Additional file 5 — Glycine riboswitches and their regulated functions identified in three metagenomes. New functions are set in boldface. [file 1471-2164-8-347-S5.pdf]

Protein function		Gene	Number of riboswitches in metagenomes (grouped by taxonomy)					
			Sargasso Sea		Minnesota Soil		Whale Falls	
Glycine cleavage system T protein (aminomethyl-transferase) (COG0404)		*gcvT*	α-Proteobacteria	59	α-Proteobacteria	2	α-Proteobacteria	3
			β-Proteobacteria	1			γ-Proteobacteria	3
			γ-Proteobacteria	1			β-Proteobacteria	1
**Malate synthase (COG2225)**		***glcB***	α-Proteobacteria	22		-		-
			γ-Proteobacteria	2		-		-
Glycine/D-amino acid oxidase (deaminating) (COG0665)		*dadA*	α-Proteobacteria	2		-	β-Proteobacteria	1
Na+/alanine symporter (COG1115)		*alsT*	γ-Proteobacteria	1		-		-
Glycine cleavage system protein P (pyridoxal-binding), C-terminal domain (COG1003)		*gcvP*	β-Proteobacteria	1	γ-Proteobacteria	1		-
L-serine deaminase (COG1760)		*sdaA*		-		-	Bacteria	1
**Phosphoserine aminotransferase (COG1932)**		***serC***	Bacteria	1		-		-
**Uncharacterized conserved protein (COG4198)**		-	Firmicutes	5		-		-
Unknown function	No ORF			-		-		-
	No similar proteins			-		-		-
	End of DNA fragment			11		1		-

Additional file 5: Glycine riboswitches and their regulated functions identified in three metagenomes. New functions are set in boldface.
