# Supplementary material for: Abundance and functional diversity of riboswitches in microbial communities
Source: BMC Genomics. 2007 Oct 1;8:347. doi: 10.1186/1471-2164-8-347 (PMC2211319; doi:10.1186/1471-2164-8-347)
Supplement: Additional file 6 — YKKC/YXKD riboswitches and their regulated functions identified in three metagenomes. [file 1471-2164-8-347-S6.pdf]

Protein function	Gene	Number of riboswitches in metagenomes (grouped by taxonomy)		
		Sargasso Sea	Minnesota Soil	Whale Falls
ABC-type nitrate/sulfonate/taurine/bicarbonate transport system, periplasmic component (COG0715)	*tauA*	-	-	δ-Proteobacteria 2<br>α-Proteobacteria 1
ABC-type nitrate/sulfonate/bicarbonate transport system, permease component (COG0600)	*tauC*	-	-	γ-Proteobacteria 1
Amino acid transporters (COG0531)	*potE*	-	β-Proteobacteria 1	-

Additional file 6: YKKC/YXKD riboswitches and their regulated functions identified in three metagenomes.
