# Supplementary material for: Abundance and functional diversity of riboswitches in microbial communities
Source: BMC Genomics. 2007 Oct 1;8:347. doi: 10.1186/1471-2164-8-347 (PMC2211319; doi:10.1186/1471-2164-8-347)
Supplement: Additional file 7 — YYBP/YKOY riboswitches and their regulated functions identified in three metagenomes. [file 1471-2164-8-347-S7.pdf]

Protein function		Gene	Number of riboswitches in metagenomes (grouped by taxonomy)					
			Sargasso Sea		Minnesota Soil		Whale Falls	
Predicted membrane protein (COG2119)		-		-	β-Proteobacteria	1	α-Proteobacteria	1
Predicted membrane protein (COG1971)		-	γ-Proteobacteria	2		-		-
Membrane protein TerC, possibly involved in tellurium resistance (COG0861)		*terC*		-	Bacteria	4		-
Unknown function	No ORF			-		1		2
	No similar proteins			-		-		-
	End of DNA fragment			-		-		-

Additional file 7: YYBP/YKOY riboswitches and their regulated functions identified in three metagenomes.
