# Supplementary figures and images for: Abundance and functional diversity of riboswitches in microbial communities
Source: BMC Genomics. 2007 Oct 1;8:347. doi: 10.1186/1471-2164-8-347 (PMC2211319; doi:10.1186/1471-2164-8-347)

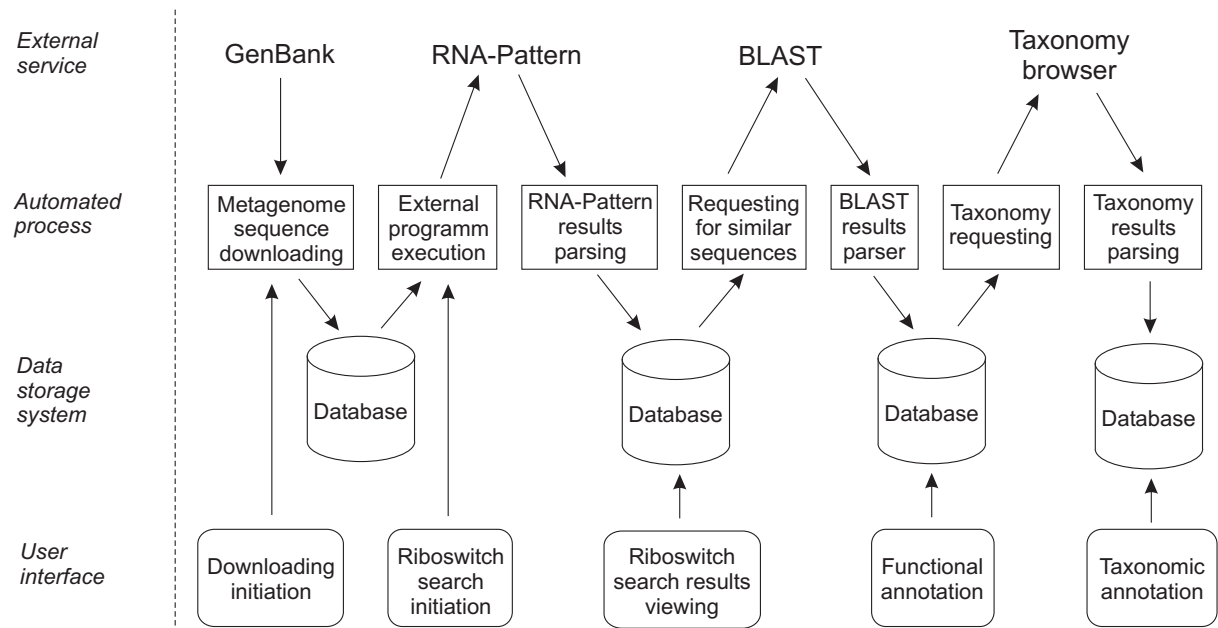

Additional file 8: Data processing flow

Supplement: Additional file 8 — Data processing flow. [file 1471-2164-8-347-S8.pdf]
