# Supplementary material for: Abundance and functional diversity of riboswitches in microbial communities
Source: BMC Genomics. 2007 Oct 1;8:347. doi: 10.1186/1471-2164-8-347 (PMC2211319; doi:10.1186/1471-2164-8-347)
Supplement: Additional file 10 — Search pattern and sequence alignment of FMN-riboswitches. [file 1471-2164-8-347-S10.pdf]

A

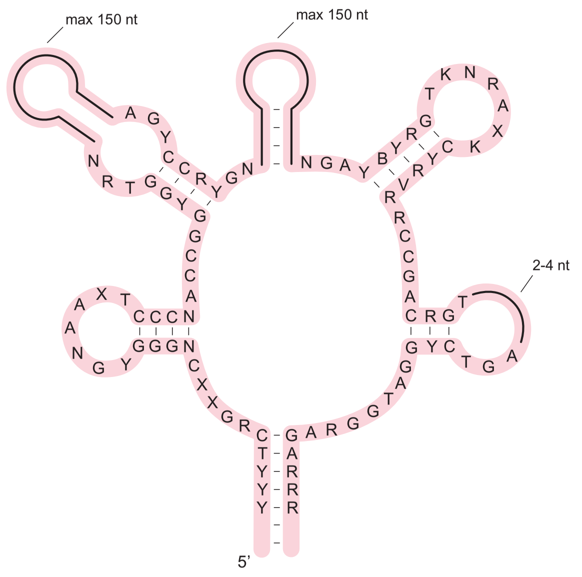

B

Accession	Metagenome	Start position	End position	Regulated function (COG)
CH073239	Sargasso	5397	5561	COG1985
CH160583	Sargasso	4674	4522	COG0307
AAFX01084096	Soil	136	295	COG0054
AAFX01111890	Soil	556	398	COG0108
AAFY01011024	Whale1	860	702	COG0108
AAFZ01027387	Whale2	32	168	COG0054
AAGA01005017	Whale3	474	311	COG0054

C

```
Structure
CH073239        CTTACTGCTAACTTCAGGGCGGGGTGAAACTCCCCACCGGCGGTGATTGTGAACTTAACCATTCGCACAAGCCCGCGAGCGCTGTT
CH160583        AAGTACATAGTCCTCGGGGTTGGGTGAAAATCCCTACCGGCGGTGACTGTTAA---------TAACAGCAGCCCGCGACCCTTGTG
AAFX01084096    GGCGGCCGCGTTCTCAGGGCGGGGTGAAAGTCCCCACCGGCGGTAATGCCTGCGCA----GGCAGGACGAGCCCGCGGGCGCCCCT
AAFX01111890    CATTGACTTATTCTCAGGTCGGGGTGAAAATCCCCACCGGCGGTATCCCAGGTTA------TACTGGGGAGCCCGCGAGCGCTCCT
AAFY01011024    TTCCGTGGAATTCTCAGGGCGGGGTGAAATTCCCCACCGGCGGTGATTTTGATGC-----AGTCAAATAAGCCCGCGAGCGCCTGT
AAFZ01027387    GTAAAATCCATTCTCAGGNCGGGGTGAAA-TCCCCACCGGCGGTTC-----------------------AGCCCGCGAGCGCCATC
AAGA01005017    CGTGAACGTATTCTCAGGGCGGGGTGCAATTCCCCACCGGCGGTATGTGGCCTT-------GCGCCACCAGCCCGCGAGCGCAACC

Structure
CH073239        AGAAAC------CTAACAGGTCAGCAGATCTGGTGTAACTCCAGAGCCGACGGTTATAGTCCGGATGCAAGAAGTATTAAGTTGA
CH160583        AAAA----------TTGATCAAGGTTGATCAGGTGAAATTCCTGAGCCGACGGTAAGAGTCCGGATGGGAGAGGATGTAAGACTG
AAFX01084096    CTCC--------GGAGGGGACAGCAGACACCGGTGCGATCCCGGGGCCGACGGTTAGAGTCCGGTTATGAGAACCGCTGCAGGCG
AAFX01111890    TCGATT------GATGGAGGTCAGCAGATCTGGTGAAAAACCAGAGCCGACGGTCACAGTCCGGATTGAAGAGGATAAGGCTGCC
AAFY01011024    TGCTT-------TAACAGGGTCAGCAGATCTGGTGAAACTCCAGAGCCGACGGTGAAAGTCCGGATGGGAGAGAACAACAGGCTG
AAFZ01027387    TTCG---------GGTGGGTCAGCAGACACTGGTGGAACTCCAGGGCCGACGGTTAGAGTCCGGATGGTAGAGAATGGGTGTGAG
AAGA01005017    ACCCCAAACAGGGCGGTTGGTCAGCAGATCTGGTAAGATGCCAGAGCCGACGGTCATAGTCCGGATGGAAGAGAATGCGTTTGTT
```

Additional file 10: (A) FMN-riboswitch (RFN-element) pattern. (B) List of identified FMN-riboswitches. (C) Alignment of FMN-riboswitch sequences.
