# Supplementary material for: Abundance and functional diversity of riboswitches in microbial communities
Source: BMC Genomics. 2007 Oct 1;8:347. doi: 10.1186/1471-2164-8-347 (PMC2211319; doi:10.1186/1471-2164-8-347)
Supplement: Additional file 11 — Search pattern and sequence alignment of cobalamin riboswitches. [file 1471-2164-8-347-S11.pdf]

Additional file 11: (A) Cobalamin riboswitch (B_{12}-element) pattern. (B) List of identified cobalamin riboswitches. (C) Alignment of cobalamin riboswitch sequences.

A

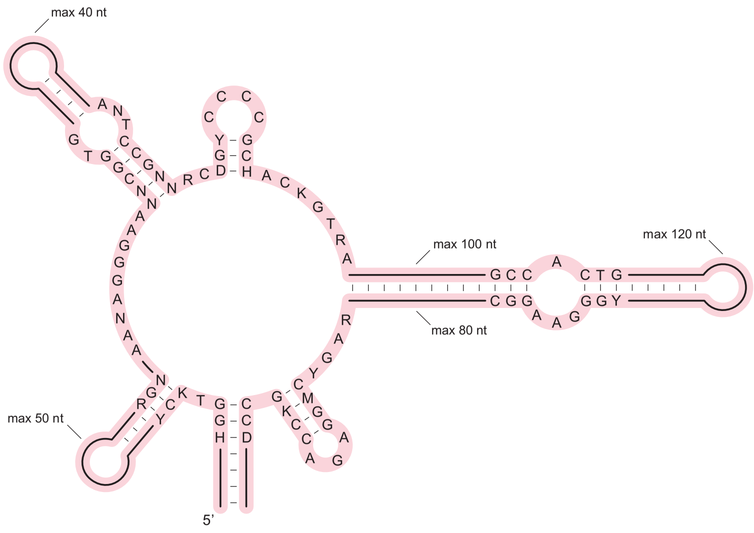

B

Accession	Metagenome	Start position	End position	Regulated function (COG)
CH004480	Sargasso	71781	71566	COG4206
CH004619	Sargasso	21809	21991	End of DNA fragment
CH004714	Sargasso	46521	46684	End of DNA fragment
CH004714	Sargasso	48933	49096	COG4206
CH007532	Sargasso	2636	2844	COG4206
CH021003	Sargasso	1469	1287	COG4206
CH024032	Sargasso	4608	4785	COG4206
CH025096	Sargasso	9886	9718	COG4206
CH026647	Sargasso	1046	1284	COG4206
CH026903	Sargasso	8001	8179	COG4206
CH026979	Sargasso	21889	21713	COG4206
CH027146	Sargasso	62416	62254	COG4206
CH027167	Sargasso	10637	10799	COG4206
CH068215	Sargasso	5472	5674	No ORF
CH079196	Sargasso	818	645	COG4206
CH082828	Sargasso	5186	5352	COG4206
CH090973	Sargasso	437	605	No ORF
CH114488	Sargasso	61	220	No similar proteins
CH118992	Sargasso	437	242	No similar proteins
CH122549	Sargasso	675	866	End of DNA fragment
CH132537	Sargasso	496	679	No ORF
CH133412	Sargasso	2948	3149	No similar proteins
CH139271	Sargasso	3396	3193	COG2370
CH140012	Sargasso	2615	2780	COG4206
CH154921	Sargasso	4533	4331	No similar proteins
CH164044	Sargasso	3817	4023	COG0523
CH167673	Sargasso	4724	4566	COG4206
CH194864	Sargasso	2225	2025	COG2370
CH208519	Sargasso	1956	2105	COG4206
CH223512	Sargasso	2352	2535	COG0523
CH228305	Sargasso	2359	2527	No similar proteins
CH233447	Sargasso	523	359	No similar proteins
AAFX01002834	Soil	203	399	COG4206
AAFX01019365	Soil	583	778	No similar proteins
AAFX01031697	Soil	761	945	No similar proteins
AAFX01036690	Soil	1213	1002	COG4206
AAFX01047844	Soil	1	146	COG0614
AAFX01048875	Soil	911	711	COG0614
AAFX01049373	Soil	234	52	No similar proteins
AAFX01053855	Soil	601	784	No similar proteins
AAFX01075090	Soil	545	788	No ORF
AAFX01087041	Soil	422	259	COG1398
AAFX01090988	Soil	145	326	No similar proteins
AAFX01091066	Soil	567	810	No ORF
AAFX01096519	Soil	189	420	COG4206
AAFX01114101	Soil	504	747	No ORF
AAFY01001147	Whale1	1565	1364	COG0614
AAFY01001400	Whale1	406	604	COG4206
AAFY01002311	Whale1	611	779	COG4206
AAFY01004391	Whale1	823	621	COG4206
AAFY01006349	Whale1	761	584	COG0609
AAFY01008015	Whale1	417	229	COG2087
AAFY01018715	Whale1	722	551	No similar proteins
AAFY01020561	Whale1	336	140	No similar proteins
AAFY01026153	Whale1	291	134	No similar proteins
AAFZ01000982	Whale2	903	704	COG4206
AAFZ01010461	Whale2	175	348	COG2087
AAGA01000085	Whale3	2922	3132	COG4206
AAGA01006787	Whale3	585	374	COG1629
AAGA01012622	Whale3	428	231	No similar proteins
AAGA01023168	Whale3	337	509	COG0620

```
Structure

CH004480      CAACCTTCTGTGGTGCTTGCGATGATG---------------AAGTCGCGAGATAATCGGGAA-GCCAGTGA-------------------------AATTCTGGCACTGC-CCCCGCAACGGTAAAAGGTGAGAGACGACCGCAT-------------------
CH004619      TATGATTGTAGGGTTTTCAGATTTAT-------------------CTAAAATTAAAACGGGAA-TCTGGTGAATTT------------------TTAGAATCCAGAACTGC-CCCCGCAACTGTAATTGGCGAGCCT----------------------------
CH004714      AAAGACCAATTGGTGCCGTTTAAT-----------------------ATCGGATAATAGGGAA-AAAGGTTT-------------------------AAATCCTTTGCTGT-ACCCGCAACTGTTATTAAGTGTTT-----------------------------
CH004714      AAAGACCAATTGGTGCCGTTTAAT-----------------------ATCGGATAATAGGGAA-AAAGGTTT-------------------------AAATCCTTTGCTGT-ACCCGCAACTGTTATTAAGTGTTT-----------------------------
CH007532      GTATTAACTACGGCGACAAGA------------------------------GTTAAACGGGAA-ATCAGTGA-------------------------AAAACTGATGCTGC-CCCCGCAACGGTAAACTCAACGCGTTGTC------------------------
CH021003      AAAATTAATTAGGTGCCTATTTTT------------------------TAGGTGAAAAGGGAA-TGAGATATTTTTT---------------ATAAATTGCCTCAGCTGC-CCCCGCAACTGTTTTTGACGAGAG------------------------------
CH024032      ATCTTTCGATGAGTTCAGCAA---------------------------ATTGCTGTTAGGGAA-GTTGGTGAA------------------------GAATCCAACACTGC-CCCCGCAACGGTGATCAAGTGAGCTGATCTT----------------------
CH025096      TAATCGACCATGGTGCCTTAAT---------------------------AGGTTAAACGGGAA-ACTGGTGAGTA--------------------AATAATCCAGTGCTGC-CCTCGCAACGGTAAATAGAAGATT-----------------------------
CH026647      GACAGTGTGTAGGTGCTCTTGTCTAGCTCTATATTAACTACGGCGACAAGGGTTAAACGGGAA-ATCAGTGA-------------------------AAAACTGATGCTGC-CCCCGCAACGGTAAACTCAACGCGTTGTC------------------------
CH026903      CTCTTACGATGAGTTCAGCAA---------------------------ATTGCTGTTAGGGAA-GTTGGTGAA------------------------GAATCCAACACTGC-CCCCGCAACGGTGATCAAGTGAGCTGATCTT----------------------
CH026979      CTCATACGATGAGTTCAGCAA---------------------------ATTGCTGTTAGGGAA-GTTGGTGAA------------------------GAATCCAACACTGC-CCCCGCAACGGTGATCAAGTGAGCTGATCTT----------------------
CH027146      TTAAAATTATTGGTGCCGCTTAAA-----------------------CACGGATAATAGGGAA-AAAGGTTT-------------------------AAATCCTTTGCTGT-ACCCGCAACTGTTTTTGAGTGTTT-----------------------------
CH027167      GTTACACCATTGGTGTTTGCAAA--------------------------AGCTTAAAAGGGAA-GTTGGTGT-------------------------AAGTCCAACACAGA--CCCGCTGCTGTAATAAGCTAATACT---------------------------
CH068215      TCGTCCGAAAAGGTTTTAAAGCAAGGACTAGAAGA--AAGATAGCCTCGCTGAAAAAAGGGAA-TCTCGTGA-------------------------AATTCGGGAACTAT-CCTCGTAACTGTAATTGAAAAAAT-----------------------------
CH079196      TTTTTCTGTTTGGTGTGCGTAAT-------------------------GCACATAAAAGGGAATCACGAAAA------------------------TTTAACGTGAGCTGCCCTCTGCAACTGTAAGCAGTGAGCCA----------------------------
CH082828      AATATACATTTGGTGTTCTGTAAA-----------------------AAGAAGTAAAAGGGAA-TATGGTTT-------------------------AAATCCATAACTGT-CTCCGCAACTGTAAATGGTGCAAAG----------------------------
CH090973      ATAACAATATTGGTTCACTGAAA------------------------AGTGATTAATAGGGAA-TACGGTTT-------------------------AAATCCGTAGCTGT-TCCCGCAACTGTAAGCAAGGAGCTT----------------------------
CH114488      GTATTGAATTTGGTGCAATT--------------------------AATTGCATAATAAGGAA-TCCGGTGT-------------------------AATTCCGGAGCTGT-ATCTGCAGCTGTAAGTATGTTAATT----------------------------
CH118992      TCAAAAACTAAGGTTTAATCTATTGCGATGAG--------TTCAGCAAATTGCTGTTAGGGAA-GTTGGTGA-------------------------GAATCCAACACTGC-CCCCGCAACGGTTATCAAGTGAGCTGATCTT----------------------
CH122549      TGAAAGTATTAGGTGCTAACTGTTTAATTTA----------TAGTAGTTTAGTTAAACGGGAA-GTTGGTCA-------------------------AATTCCAACGCTGC-CCCCGCAACGGTAGATGAGTTAGAG----------------------------
CH132537      CAGATAGATGTGGTCCTCCAC--------------------------TGGAGGTAATAGGGAA-TGTGGTAACGCCATCA----------TTATGGCAATGCCGCAGCTGC-TCCCGCAACTGTGTGCTGTTTGTGCG---------------------------
CH133412      GGGTCCGAAAAGGTTTTACTGAAGGGACCACAAG---ACAGGTAACTTTGCTGAAAAAGGGAA-TCTCGTGA-------------------------AATTCGGGAACTAT-CCTCGTAACTGTAATTGAAAAAGAT----------------------------
CH139271      TATTGGAAGTAGGTGGCTGTGATGCACAG-------------CGTCATGCCTTAATGAGGGAA-TAGGGTAAGCATGAAAC-------AAGCCATGTAAAGCCCTAGCCGC-CCCCGCGACTGTAAGTTGGTCGTT-----------------------------
CH140012      TTAATGACGTTGGTTCACGTAAA-------------------------GTGAATAACAGGGAA-TAAGGTTT-------------------------AAATCCTTAGCTGT-ACCCGCAACTGTAACTCATGAGTTT----------------------------
CH154921      TTCCCTGAAGAGGTTTTACTGCAGGGACTAGAAGA--CAGATAACCTTGCTGAAAAAAGGGAA-TCTCGTGA-------------------------AATTCGGGAACTAT-CCTCGTAACTGTAATTGAAAAAGATAAAAGC----------------------
CH164044      AACAAGTCTACGGTTCCTAATTT-------------------------GGATTAAAAAGGGAACGTGGGTACTCGCTAATAAT---TATATTGGTGAGCAGCCACGGCTGT-CCCCGCAACTGTGTGTGGTGAGCTAG---------------------------
CH167673      ----------------------------------------TGTAAAAAAATTAAAAGGGGGAA-TACGGTGTAA---------------------ATTAATCCGTAGCTGC-CCCCGCAACTGTATTTGGTTAGCTTT---------------------------
CH194864      CAATCATTTGTGGTTCTGCTGTACAACGAG-----------TGAACAGCAGATTAAGAGGGAA-TATGGTGAGGTGATATTA------ACAGGAATCAAGGCCATAACTGC-CCCCGCAACTGTAAGCGTGTAGCCCG---------------------------
CH208519      ---------------------------------------------------GTGAAAAGGGAA-TGAGATATTATAA--------------GTATAGTTACCTCAGCTGC-CCCCGCAACTGTATATAAGGAGAG------------------------------
CH223512      ATTTATCGATGGGTTCCCAAACAT----------------------TTTGGGATTCAAGGGAA---CGTAGTT-----------------------AGAATCTACGGCTGC-CCCCGCAACTGTAAGCGGAGAGTTTT---------------------------
CH228305      TCACTTAAATTGGTTTTTGCAATTTAT------------------AGCAAAATTAAAAGGGAA-TAATGTGT-------------------------GAATCATTAACTGT--CCCGCAACTGTAATTTGATAACATT---------------------------
CH233447      GTTTCAATGTTGGTTCACGTAAA-------------------------GTGAATAACAGGGAA-TAAGGTTT-------------------------AAAGCCTTAACTGT-ACCCGCAACTGTAACTCTGGAATTT----------------------------
AAFX01002834  GTGAGTGTGCTGGTTCCCCGGTTCG-----------------CGCCGGGGAGGTAAGAGGGAA-CACGGGCAGGCTGCCGT-------TTGCAGTCTTCATCCGTGGCTGC-CCCCGCAACTGTAAGCGGCGAGCCTG---------------------------
AAFX01019365  CATCTCGTGTTGGTGCACTGGGCATC----------------TGCCCAGGGCTGAAAAGGGAA-CCCGGTGA-------------------------AATTCCGGGGCTGC--CCCGCAGCGGTAAGCGAGAACGAG----------------------------
AAFX01031697  TGAACGCCTCGGGTGCCCATGAG------------------------GGGCATGACAGGGAAGACCGGTGA--------------------------GAATCCGGCGCGGT--TCCGCCACTGTGAGTGAGGGCGCGTCGCTTC---------------------
AAFX01036690  ---------AGTGCGCCCCGCCG--------------------------AAAGGTAACGGGAA-GCTGGTGAGCCGCGAGA--------CAGCGCGCGATTCCAGCACTGC-CCCCGCAACGGTAATCGAACCAGAAACCTGC----------------------
AAFX01047844  ------------------------------------------------------GCGCGGGAA---CGGTAGCCGG--------------------TGTGATACCGGCGCGGC--CCCGCCACTGTAACGGGCGTTCTAC-------------------------
AAFX01048875  CGAAAGTTTTGGGTGTCGCGCGTTTTCA---------TAGAAAACGCGCGAAGAATAGGGAAGTCGGGTGAA--------------------------AATCCCGCACGGT--CGCGCCACTGTGAACGGATTTTAGATTTTAGATTTTAGATTTT---------
AAFX01049373  GCACCGTATTTGGTTCTTGTACATCAGCAA----------GATGGACAAGATTAAAAGGGAATACCGGTGA--------------------------GAATCCGGAGCAGT-TCCCGCTACTGTAAATACTGTTAAGCGT-------------------------
AAFX01053855  ACCGAGGTTTCGGTTCCGAATGTTTTG------------------GAACCGGAGAATAGGGAA-TACGGTGT-------------------------GAATCCGTAACAGT-ACCCGCTGCTGTATGCCGGCGCTCCTTGTGGAG--------------------
AAFX01075090  TTCTCCGATTTGGTCCCCGCATG---------------------TGCGGGGTTAAAAGGGAAGTCCGGTGT--------------------------AATTCCGGCGCTAT-CCCCGTAGCTGTAAGGCAGTGAATGGTCAATGAGTGGTGAATCGATTCTGAAT
AAFX01087041  CAGGCGCCCGGGTGCCCATA------------------------------CGGGCCCAGGGAA-GGCGGTGC-------------------------GAATCCGCCGCGAC--CCCGTCACTGTAACCGGGGACGAA----------------------------
AAFX01090988  AAGCGCCGCGTGGATAC-------------------------------------GGAAGGAAA-GCCGGTGT-------------------------GAGTCCGGCGCGGT--CCCGCCACTGTAAACGGTGAGGTGCCGTCCAGAAGCT---------------
AAFX01091066  TTCTCCGATTTGGTCCCCGCATG---------------------TGCGGGGTTAAAAGGGAAGTCCGGTGT--------------------------AATTCCGGCGCTAT-CCCCGTAGCTGTAAGGCAGTGAATGGTCAATGAGTGGTGAATCGATTCTGAAT
AAFX01096519  GCCCCGTAATTGGTTTTTCTGCTTCAC----------------GCAGAGAATTAAAAGGGAAGACCGGTGT--------------------------AAATCCGGCGCTAT-CCCCGTAGCTGTAAGGTTTGTGAATGGTGAAAGAAGAATGGTGAATAATTT--
AAFX01114101  TTCTCCGATTTGGTCCCCGCATG---------------------TGCGGGGTTAAAAGGGAAGTCCGGTGT--------------------------AATTCCGGCGCTAT-CCCCGTAGCTGTAAGGCAGTGAATGGTCAATGAGTGGTGAATCGATTCTGAAT
AAFY01001147  AATTGATTATCGGTATCCGAAA--------------------------TTCGGATAAAGGGAA-TTCGAAACCGATTGCCCCGTCTGGGGCATTTGGTGAACCGAAGCTGC-CCCCGCAACTGTAGGCGGTGAGCATTA--------------------------
AAFY01001400  GGCAGATATCGGGTTTCTCTTAATC----------------------GAGAATTCAAAGGGAA-ATCGGTGAGGAAAGGGTTT---GCGCCCGTTCCAAATCCGGTACTGC-CCCCGCAACTGTAAGCGGCTAGCTG----------------------------
AAFY01002311  GACATAGTTAGGGCTTTT-----------------------------GCCATTTAATAGGGAA-TCCCGTGG-------------------------AAATCGGGAACGGA--CCCACCGCTGTAACCGGGGACAAA----------------------------
AAFY01004391  GTTTCGTATTTGGTTTTACATTTAATTTT--------------AAATGTGAATTAAAAGGGAA-TTCTGTTA-------------------------AAATCAGAAGCTGT-TCCCGCAACTGTAAGCTCAGTCTTTATAGAAAAGATGC---------------
AAFY01006349  GGATATTTTCGGGGGCTGCCCATA-----------------------GGCAGTTAAAGGGGAA-TCCTGTGA-------------------------AATTCAGGAACGGG-CCCCGCCGCTGTGACCGGGGATGAA----------------------------
AAFY01008015  CTGATAGGTTCGGTTTTGCCGT----------------------------TTGGTGATGGGAA-ATCGGTGT-------------------------GATGCCGATGCTGC-CCCCGCAACTGTAACGGGTTGGGTGATCTAG----------------------
AAFY01018715  GTTGTGTTATTGGTTTTCAGATTTAAA-----------------GTCTGGAATTAATAGGGAA-TCAGGTGA-------------------------AAAACCTGAACTGT-ACCCGCAACTGTAAACGCTGTAAGT----------------------------
AAFY01020561  ATGCTCACTGAGGTGTCGCGTGATTGAT---------------ACACGCG-ATTAAACGGGAA-ACAGGTGCTGACAC-------------CCGTCAAAAGCCTGTGCTGC-CCCCGCAACGGTAAGATGTTGTC------------------------------
AAFY01026153  ATTAGATCGGCAGCGTC----------------------------------------AGGGAA-GCAGGTTA-------------------------AATTCCTGCGCGGC--CCCGCCGCTGTAACCGGAAACGA-----------------------------
AAFZ01000982  CGCCACCTTTCGGTGCCCACCCTGAACA---------------CAGGGCGGGTTAAACGGGAA-GCCGGTGAGAGCCT-------------TGGCTCTAATCCGGCGCTGC-CCCCGCAACGGTGATTGAGTAAACG----------------------------
AAFZ01010461  CAAAGCTGTTAGGTTCGGCGCACCGT------------------------TCGGTGATGGGAA-ATCGGTGA-------------------------AATGCCGATGCTGC-CCCCGCAACTGTATCGGGTTGGGCGATCCG-----------------------
AAGA01000085  CGCTGAATTTGGTTTGACTTTGTGCT-------------AATGCAAAGTCGATTAAAAGGGAA-TCTGGTTT-------------------------AAATCCAGAACTGT-TCCCGCAACTGTAAGTTTATGTTCAACATTAGTTGGACACC------------
AAGA01006787  CATCGATTTTTGGTTTGATGTTATTTTTT-------------TAACATTGAATTAAAAGGGAA-TCTGGTAA-------------------------AAAGCCAGAACTGT-TCCCACAACTGTAAGTTTATGCTGATATTTTTTCAGCACC-------------
AAGA01012622  GCCAGATATTGGGTTTCTCTTAATC----------------------GAGAATTCAAAGGGAA-ATCGGTGAGAAAAGGCTTC----GGCCCGATTCAAATCCGGTACTGC-CCCCGCAACTGTAAGCGGCTAGCCG----------------------------
AAGA01023168  TCAAACAGATAGGTTCTACCTA-----------------------AGGTAGATTAATAGGGAA-TCGTGTGC-------------------------AAATCACGAACTGT--CGCGCAACTGTAAGTAACAATTTAAGGG------------------------
```

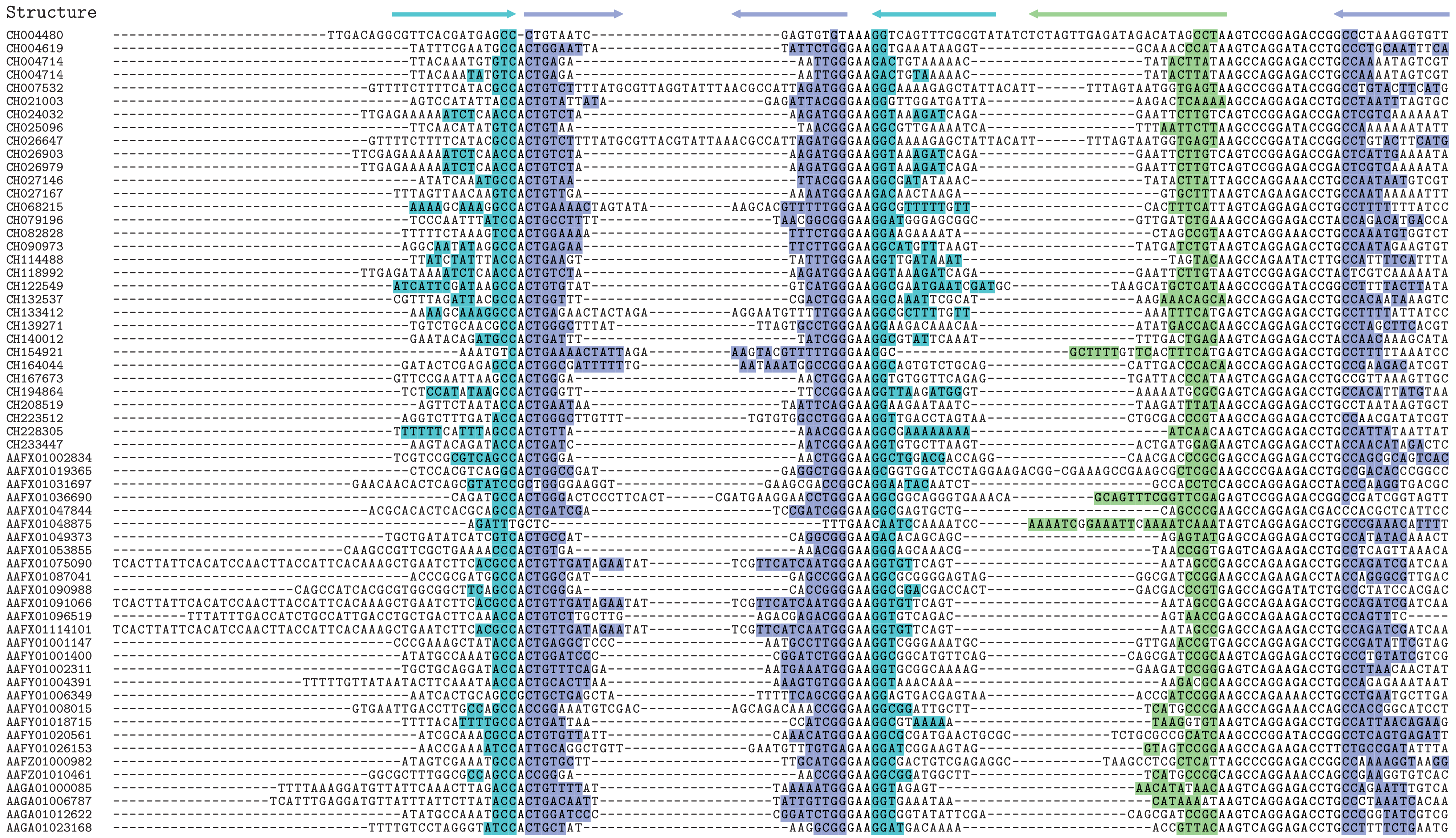
Structure
CH004480
CH004619
CH004714
CH004714
CH007532
CH021003
CH024032
CH025096
CH026647
CH026903
CH026979
CH027146
CH027167
CH068215
CH079196
CH082828
CH090973
CH114488
CH118992
CH122549
CH132537
CH133412
CH139271
CH140012
CH154921
CH164044
CH167673
CH194864
CH208519
CH223512
CH228305
CH233447
AAFX01002834
AAFX01019365
AAFX01031697
AAFX01036690
AAFX01047844
AAFX01048875
AAFX01049373
AAFX01053855
AAFX01075090
AAFX01087041
AAFX01090988
AAFX01091066
AAFX01096519
AAFX01114101
AAFY01001147
AAFY01001400
AAFY01002311
AAFY01004391
AAFY01006349
AAFY01008015
AAFY01018715
AAFY01020561
AAFY01026153
AAFZ01000982
AAFZ01010461
AAGA01000085
AAGA01006787
AAGA01012622
AAGA01023168
