# Supplementary material for: Abundance and functional diversity of riboswitches in microbial communities
Source: BMC Genomics. 2007 Oct 1;8:347. doi: 10.1186/1471-2164-8-347 (PMC2211319; doi:10.1186/1471-2164-8-347)

Additional file 12: (A) TPP-riboswitch (THI-element) pattern. (B) List of identified TPP-riboswitches. (C) Alignment of TPP-riboswitch sequences.

A

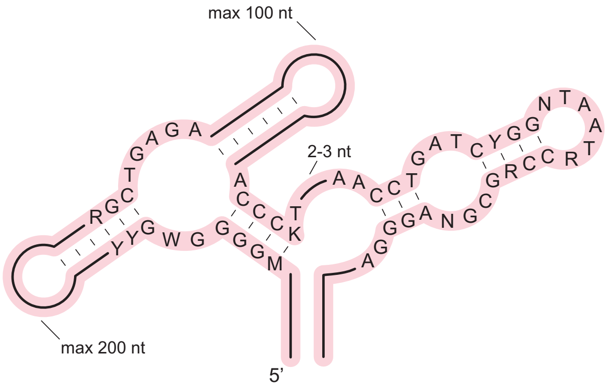

B

Accession	Metagenome	Start position	End position	Regulated function (COG)
CH004445	Sargasso	154176	154084	COG0422
CH004672	Sargasso	10398	10490	COG1629
CH004725	Sargasso	369227	369337	COG0422
CH005713	Sargasso	2511	2408	End of DNA fragment
CH007820	Sargasso	27596	27690	COG1116
CH009295	Sargasso	1	49	COG0600
CH013922	Sargasso	3521	3605	COG4143
CH015221	Sargasso	580	663	COG0422
CH016634	Sargasso	8974	9067	COG0591
CH016915	Sargasso	1268	1228	COG4143
CH017495	Sargasso	441	350	COG4143
CH018943	Sargasso	21448	21356	COG1629
CH020882	Sargasso	7935	7836	COG1629
CH021838	Sargasso	3472	3431	COG1629
CH022308	Sargasso	6591	6705	COG1629
CH023040	Sargasso	553	469	COG4143
CH024732	Sargasso	6486	6577	COG0591
CH024980	Sargasso	8541	8450	COG4143
CH024980	Sargasso	10701	10610	COG4143
CH025245	Sargasso	8867	8770	COG1629
CH025757	Sargasso	22470	22376	COG4143
CH025757	Sargasso	22899	22805	COG4143
CH026934	Sargasso	17591	17494	COG1629
CH027395	Sargasso	7802	7895	COG4143
CH027395	Sargasso	9652	9745	COG4143
CH069812	Sargasso	5745	5662	COG0715
CH074105	Sargasso	5496	5580	COG2145
CH090651	Sargasso	1674	1769	End of DNA fragment
CH095282	Sargasso	1621	1716	COG1629
CH100699	Sargasso	251	168	COG4143
CH102139	Sargasso	380	474	COG4143
CH105054	Sargasso	6438	6333	No similar proteins
CH112697	Sargasso	461	377	COG4143
CH118322	Sargasso	3515	3472	COG0422
CH124133	Sargasso	2679	2585	End of DNA fragment
CH125213	Sargasso	889	973	End of DNA fragment
CH128347	Sargasso	496	580	COG4143
CH128607	Sargasso	563	468	COG4143
CH142156	Sargasso	276	182	No ORF
CH145540	Sargasso	169	263	COG4143
CH146210	Sargasso	783	875	End of DNA fragment
CH150887	Sargasso	244	160	No similar proteins
CH151067	Sargasso	3869	3948	COG4143
CH153908	Sargasso	717	807	No similar proteins
CH156858	Sargasso	4043	3945	End of DNA fragment
CH162170	Sargasso	482	582	COG1116
CH162897	Sargasso	630	533	COG1629
CH165163	Sargasso	543	646	No similar proteins
CH165327	Sargasso	4290	4382	No similar proteins
CH165613	Sargasso	4141	4238	COG1629
CH167172	Sargasso	4387	4304	COG4143
CH174895	Sargasso	1575	1495	COG0715
CH175023	Sargasso	221	331	COG0422
CH190652	Sargasso	537	430	COG0422
CH203500	Sargasso	1658	1561	No similar proteins
CH207169	Sargasso	2724	2808	End of DNA fragment
CH215308	Sargasso	581	676	No similar proteins
CH215423	Sargasso	180	83	End of DNA fragment
CH219306	Sargasso	265	184	No similar proteins
CH235848	Sargasso	8800	8714	COG2145
AAFX01021754	Soil	661	781	No similar proteins
AAFX01034377	Soil	335	243	COG4143
AAFX01035049	Soil	959	864	COG2145
AAFX01036045	Soil	1	102	COG4143
AAFX01041345	Soil	837	723	COG0422
AAFX01053962	Soil	1	96	COG1564
AAFX01071003	Soil	651	747	No similar proteins
AAFX01073425	Soil	342	459	COG0422
AAFX01083098	Soil	202	303	COG1629
AAFX01088025	Soil	610	511	COG0422
AAFX01095544	Soil	899	857	COG0422
AAFX01112544	Soil	1	98	COG1564
AAFX01125019	Soil	675	593	COG0422
AAFX01130813	Soil	432	331	COG0422
AAFY01006381	Whale1	486	577	COG4143
AAFY01012408	Whale1	846	944	End of DNA fragment
AAFY01013567	Whale1	764	659	COG4143
AAFY01016373	Whale1	179	74	End of DNA fragment
AAFY01023680	Whale1	440	531	COG1629
AAFZ01000566	Whale2	1779	1904	COG0422
AAFZ01014372	Whale2	646	550	COG0351
AAFZ01015815	Whale2	439	528	No similar proteins
AAFZ01018230	Whale2	435	541	COG4143
AAFZ01019953	Whale2	69	174	COG0422
AAFZ01020747	Whale2	279	185	COG0422
AAFZ01022177	Whale2	588	683	COG0422
AAFZ01022981	Whale2	582	676	COG0422
AAFZ01023302	Whale2	264	170	COG0422
AAFZ01028830	Whale2	562	471	COG0422
AAGA01003741	Whale3	1144	1236	No similar proteins
AAGA01004198	Whale3	545	445	COG4143

C

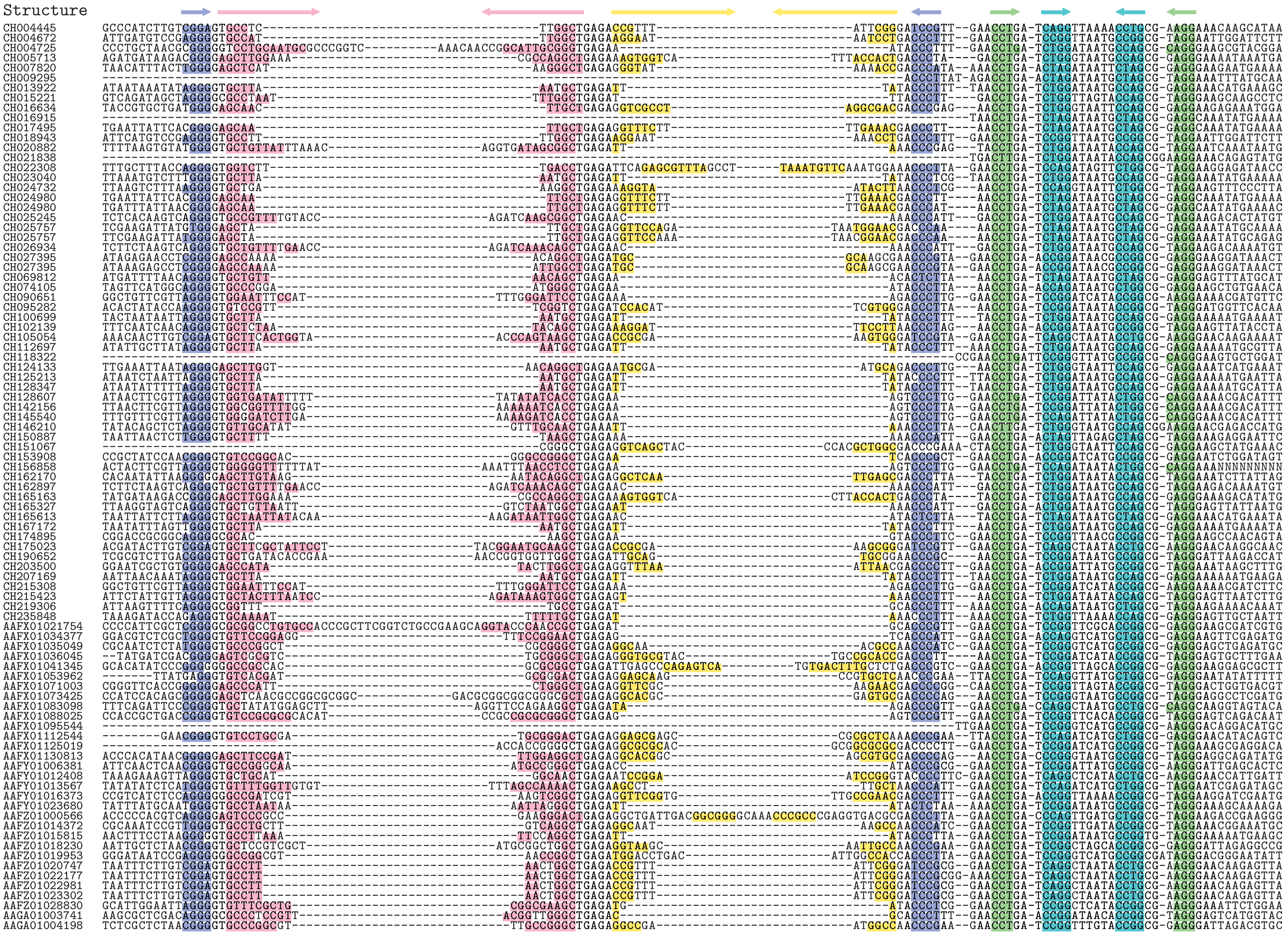

Supplement: Additional file 12 — Search pattern and sequence alignment of TPP-riboswitches. [file 1471-2164-8-347-S12.pdf]
