# Supplementary material for: Abundance and functional diversity of riboswitches in microbial communities
Source: BMC Genomics. 2007 Oct 1;8:347. doi: 10.1186/1471-2164-8-347 (PMC2211319; doi:10.1186/1471-2164-8-347)
Supplement: Additional file 13 — Search pattern and sequence alignment of glycine riboswitches. [file 1471-2164-8-347-S13.pdf]

Additional file 13: (A) Glycine riboswitch pattern. (B) List of identified glycine riboswitches. (C) Alignment of glycine riboswitch sequences.

A

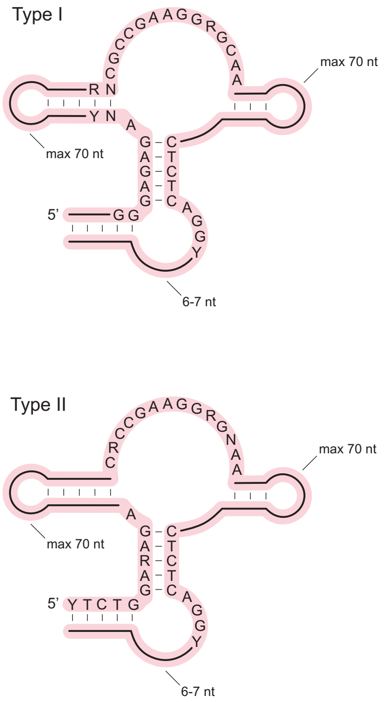

B

Accession	Metagenome	Start position	End position	Regulated function (COG)
CH004480	Sargasso	22329	22095	COG1115
CH004560	Sargasso	12265	12112	COG0404
CH004588	Sargasso	199731	199467	COG0404
CH004791	Sargasso	913	759	COG0404
CH004804	Sargasso	2864	3020	COG2225
CH006134	Sargasso	743	889	COG0404
CH007312	Sargasso	3603	3453	COG0404
CH008153	Sargasso	2710	2556	COG4198
CH010153	Sargasso	5220	5069	COG0404
CH011183	Sargasso	1755	1601	COG4198
CH011659	Sargasso	1637	1811	COG1003
CH011954	Sargasso	667	749	COG193
CH011983	Sargasso	2959	2805	COG4198
CH012769	Sargasso	2350	2201	COG0404
CH012961	Sargasso	961	1112	COG2225
CH013016	Sargasso	13773	13618	COG0404
CH013294	Sargasso	1816	1969	COG0404
CH015205	Sargasso	1386	1539	COG2225
CH016265	Sargasso	3331	3481	COG2225
CH017510	Sargasso	2573	2418	COG4198
CH017782	Sargasso	2651	2806	COG0404
CH017863	Sargasso	1451	1598	COG0404
CH018172	Sargasso	3511	3361	COG0404
CH018728	Sargasso	2076	1922	COG2225
CH020130	Sargasso	104	258	COG0404
CH020513	Sargasso	3268	3418	COG2225
CH020674	Sargasso	2952	2797	COG0404
CH020926	Sargasso	4460	4306	COG0404
CH021291	Sargasso	354	202	COG2225
CH021582	Sargasso	4258	4364	COG0404
CH021884	Sargasso	3064	3215	COG0404
CH022553	Sargasso	2666	2516	COG0404
CH023146	Sargasso	2511	2361	COG0404
CH023194	Sargasso	254	402	COG0404
CH023307	Sargasso	6379	6229	COG2225
CH023495	Sargasso	7543	7693	COG2225
CH023621	Sargasso	1239	1415	COG0404
CH024864	Sargasso	4718	4569	COG0404
CH025192	Sargasso	2898	2743	COG0404
CH025209	Sargasso	4630	4779	COG0404
CH025408	Sargasso	2036	1881	COG0404
CH025417	Sargasso	26854	26704	COG0404
CH025460	Sargasso	4746	4897	COG0404
CH025596	Sargasso	12283	12134	COG0404
CH025790	Sargasso	12265	12420	COG2225
CH027219	Sargasso	8205	8358	COG0404
CH027494	Sargasso	18921	19069	COG0404
CH027603	Sargasso	17163	17317	COG0404
CH066412	Sargasso	5792	5946	No ORF
CH071812	Sargasso	187	36	No ORF
CH073298	Sargasso	358	201	COG0404
CH074447	Sargasso	5470	5620	COG2225
CH076671	Sargasso	5548	5394	COG0404
CH078525	Sargasso	766	915	No ORF
CH082615	Sargasso	459	609	COG2225
CH084621	Sargasso	355	204	COG2225
CH085125	Sargasso	5557	5712	COG0404
CH090711	Sargasso	1266	1111	COG2225
CH095246	Sargasso	48	218	COG0665
CH099209	Sargasso	6146	6301	COG0404
CH099604	Sargasso	6282	6129	End of DNA fragment
CH107700	Sargasso	685	855	No ORF
CH108739	Sargasso	158	1	End of DNA fragment
CH110771	Sargasso	6436	6591	COG2225
CH112937	Sargasso	6223	6374	COG0404
CH113666	Sargasso	264	115	COG0404
CH113875	Sargasso	6782	6631	COG2225
CH119896	Sargasso	74	267	COG0404
CH120223	Sargasso	3370	3218	COG2225
CH123522	Sargasso	3491	3339	COG0404
CH125274	Sargasso	2650	2833	COG0404
CH127439	Sargasso	638	464	COG0404
CH127838	Sargasso	156	1	End of DNA fragment
CH128974	Sargasso	2814	2662	End of DNA fragment
CH144138	Sargasso	3958	4107	COG0404
CH145003	Sargasso	463	616	COG0404
CH147036	Sargasso	312	158	COG0404
CH148831	Sargasso	518	365	COG0404
CH149128	Sargasso	4250	4100	COG2225
CH152090	Sargasso	4328	4176	COG0404
CH153469	Sargasso	72	223	COG2225
CH160558	Sargasso	318	401	COG4198
CH161879	Sargasso	4420	4590	COG0665
CH165648	Sargasso	4282	4132	COG0404
CH167803	Sargasso	601	683	End of DNA fragment
CH169997	Sargasso	609	458	COG2225
CH172733	Sargasso	2007	1852	COG0404
CH178850	Sargasso	716	568	COG0404
CH179282	Sargasso	699	551	COG0404
CH182662	Sargasso	626	472	COG2225
CH187196	Sargasso	165	1	End of DNA fragment
CH189992	Sargasso	2154	2000	COG0404
CH192214	Sargasso	2006	1856	COG2225
CH193729	Sargasso	1596	1746	COG2225
CH193852	Sargasso	388	240	COG0404
CH193915	Sargasso	2063	1887	COG0404
CH203668	Sargasso	1821	1666	COG0404
CH205377	Sargasso	1978	1823	COG2225
CH205699	Sargasso	173	321	COG0404
CH212688	Sargasso	321	472	COG0404
CH213866	Sargasso	2294	2448	COG0404
CH215823	Sargasso	169	318	COG0404
CH217657	Sargasso	2690	2835	End of DNA fragment
CH217994	Sargasso	2104	2259	COG0404
CH230524	Sargasso	375	523	COG0404
CH234615	Sargasso	347	198	COG0404
AAFX01016906	Soil	854	674	COG0404
AAFX01042973	Soil	245	50	End of DNA fragment
AAFX01074356	Soil	708	464	COG1003
AAFX01086649	Soil	565	375	COG0404
AAFZ01013399	Whale2	650	438	COG0665
AAFZ01013783	Whale2	487	735	COG0404
AAFZ01014837	Whale2	525	718	COG0404
AAFZ01019250	Whale2	544	310	COG1760
AAFZ01024364	Whale2	419	229	COG0404
AAFZ01025915	Whale2	380	180	COG0404
AAFZ01027930	Whale2	601	401	COG0404
AAGA01003599	Whale3	265	456	COG0404
AAGA01022452	Whale3	684	470	COG0404

C

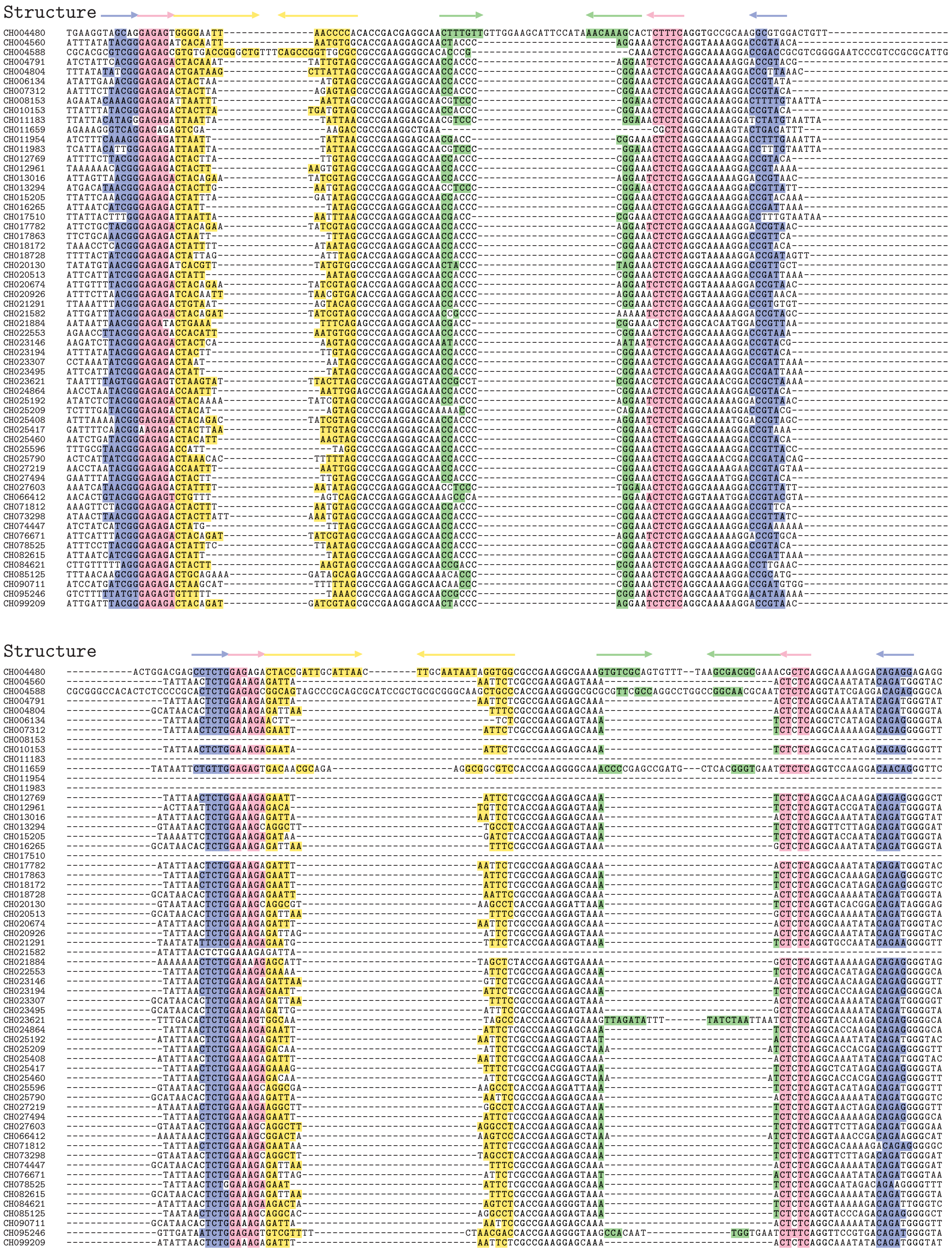

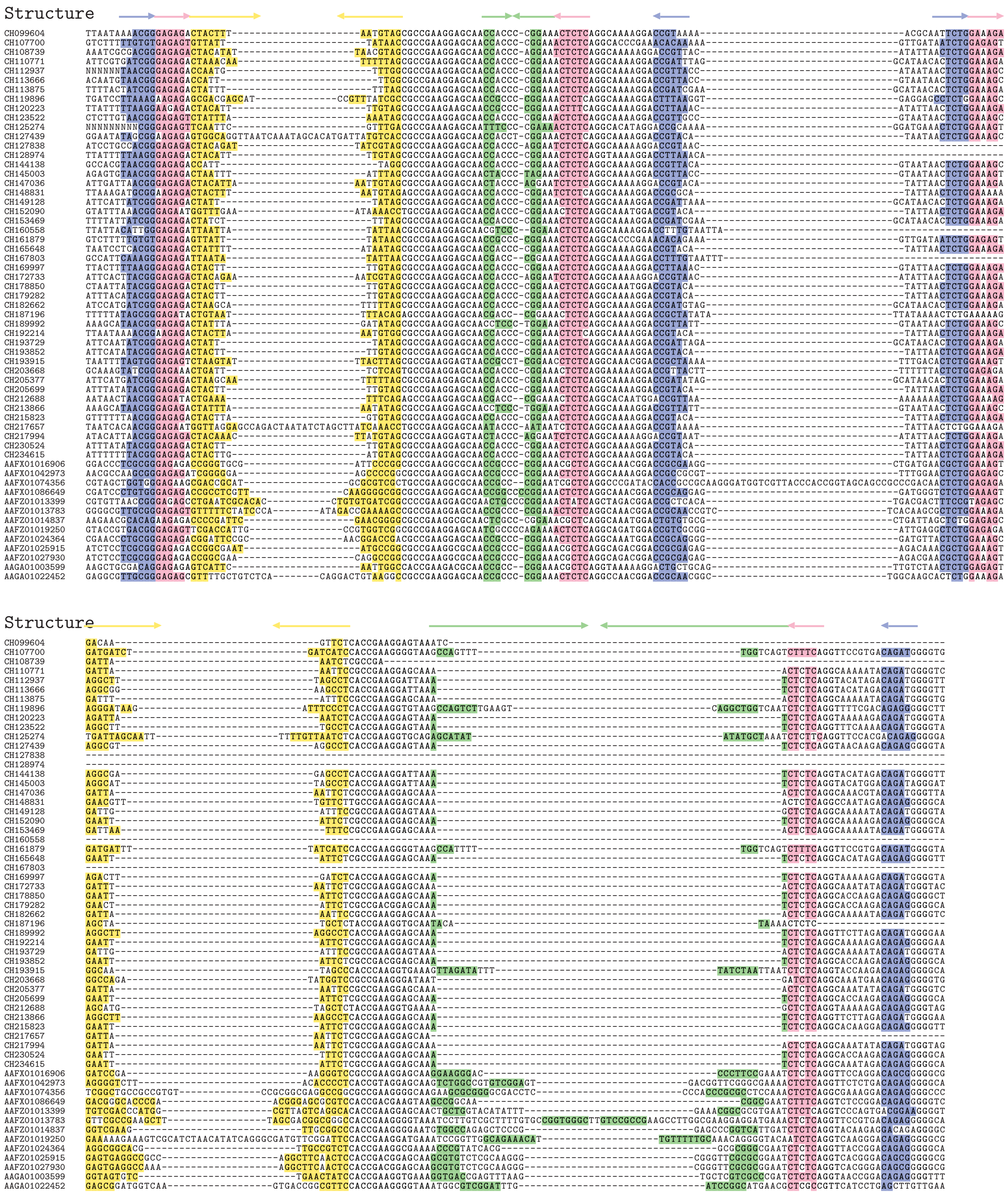
Structure
CH099604
CH107700
CH108739
CH110771
CH112937
CH113666
CH113875
CH119896
CH120223
CH123522
CH125274
CH127439
CH127838
CH128974
CH144138
CH145003
CH147036
CH148831
CH149128
CH152090
CH153469
CH160558
CH161879
CH165648
CH167803
CH169997
CH172733
CH178850
CH179282
CH182662
CH187196
CH189992
CH192214
CH193729
CH193852
CH193915
CH203668
CH205377
CH205699
CH212688
CH213866
CH215823
CH217657
CH217994
CH230524
CH234615
AAFX01016906
AAFX01042973
AAFX01074356
AAFX01086649
AAFZ01013399
AAFZ01013783
AAFZ01014837
AAFZ01019250
AAFZ01024364
AAFZ01025915
AAFZ01027930
AAGA01003599
AAGA01022452
Structure
