# Supplementary material for: Abundance and functional diversity of riboswitches in microbial communities
Source: BMC Genomics. 2007 Oct 1;8:347. doi: 10.1186/1471-2164-8-347 (PMC2211319; doi:10.1186/1471-2164-8-347)
Supplement: Additional file 14 — Search pattern and sequence alignment of YKKC/YXKD riboswitches. [file 1471-2164-8-347-S14.pdf]

A

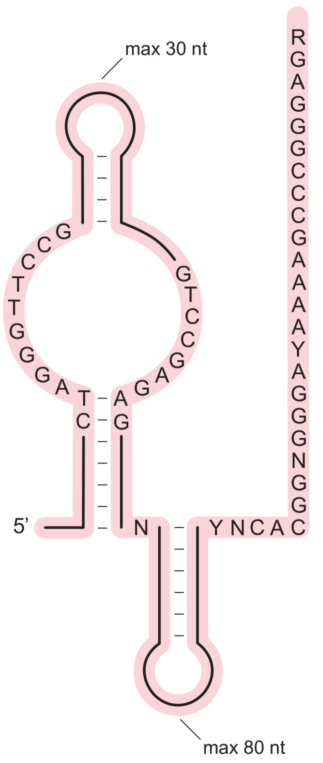

B

Accession	Metagenome	Start position	End position	Regulated function (COG)
AAFX01006062	Soil	501	390	COG0531
AAFY01000234	Whale1	1646	1540	COG0715
AAFY01010653	Whale1	806	697	COG0600
AAFY01015288	Whale1	311	420	COG0715
AAFY01021029	Whale1	845	729	COG0715

C

```
Structure

AAFX01006062   TAAAGCGAAACTAGGGTTCCGGCTTTCTGCTGAAAGTGTCTGGTCCGAGAGTTTCCGGCTTCGTC-------GTGACGAGGCTCCACGGAGGGATAAAAGCCCGGGAGATAATGTAAGT
AAFY01000234   CTAAAGGTAGCTAGGGTTCCGACTTATT--TATAAGTGTCTGGTCCGAGAGCAACCGACTCT----------TAAATAGAGTTACACGGAGGGATAAAAGCCCGGGAGGTGAATACCTC
AAFY01010653   AAATAGAGCGCTAGGGTTCCGGCTGAGCT-GTTCAGTGACTGGTCCAAGAGCACTCGACCTNT--------CAGACAAAGGTTACACGGCGGGACAAAAGCCCGGGAGACTATTGGCGA
AAFY01015288   TAATAGATAACTAGGGTTCCGGTGAATTT-ACTTCACGTCTGGTCCGAGAGTTGTCAACCTCA--------TGAGTAGAGGTCACACGGAGGGATAAAAACCCGGGAGGTTATCAACCT
AAFY01021029   GGCAGCGCGCTAGGGGTTCCGGTTCCGA--TAAGAAATGCTGGTCCAAGAGGCGCAAGCCGACCAACACATCATAGGTCGGTAACACGGCGGGCCAAAATCCCGGGAGACAACTGCACA
```

Additional file 14: (A) YKKC/YXKD riboswitch pattern. (B) List of identified YKKC/YXKD riboswitches. (C) Alignment of YKKC/YXKD riboswitch sequences.
