# Supplementary material for: Abundance and functional diversity of riboswitches in microbial communities
Source: BMC Genomics. 2007 Oct 1;8:347. doi: 10.1186/1471-2164-8-347 (PMC2211319; doi:10.1186/1471-2164-8-347)
Supplement: Additional file 15 — Search pattern and sequence alignment of YYBP/YKOY riboswitches. [file 1471-2164-8-347-S15.pdf]

A

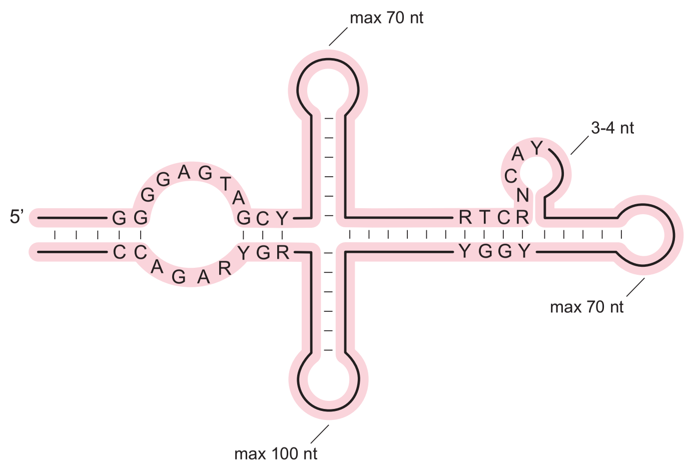

B

Accession	Metagenome	Start position	End position	Regulated function (COG)
CH025042	Sargasso	236	349	COG1971
CH027617	Sargasso	2221	2108	COG1971
AAFX01006168	Soil	410	534	COG0861
AAFX01013281	Soil	659	513	COG0861
AAFX01019015	Soil	466	328	COG2119
AAFX01069316	Soil	219	336	COG0861
AAFX01111584	Soil	309	172	No ORF
AAFX01128094	Soil	605	483	COG0861
AAFY01005645	Whale1	334	216	COG2119
AAFY01017336	Whale1	236	105	No ORF
AAFY01027355	Whale1	461	596	No ORF

C

```
Structure

CH025042      CCCCTCGGTTGGGGAGTAGCCTGTTTCCGTTTC-----------GAATGGAAAAGTTTG-TATCAACATAATTGGTC----
CH027617      CCCCTCGGTTGGGGAGTAGCCTGTTTCCGTTTC-----------GAATGGAAAAGTTTG-TATCAACATAATTGGTC----
AAFX01006168  ACATAGTGTTGGGGAGTAGCCCACCGGCGCCGGTTCG--ATGGCCGCCGTCGGTGCGACCGGTCGTCAAGACGGTGCCCT-
AAFX01013281  GAATCGAGAAGGGGAGTAGCCCACCGACCAT----------------CAGGTCGGCGGCCGAATCGTCAAGACTGCGGGCTC
AAFX01019015  TTCCTCCTCCGGGGAGTAGCCCCTCCGCGGCCGGCGTTGCCGGCATGGCGGGGACGCGC--GTCAACACGCTTGGCC----
AAFX01069316  TAGTAGGGAAGGGGAGTAGCCACGCGGGGTC--------------CACCCGCGCGGCCG-AATCGTCAAGACTGCGCG---
AAFX01111584  GAGCGTCAAAGGGGAGTAGCTACGGGGGAGCGGCAG--------GACGCCCCCGCGGCAAGGCCGTCATCACGGGATTCCG
AAFX01128094  TCTCCCCGGTGGGGAGTAGCCACCCAGGATCTC--------------TCCTGGGTCGAAGGGCCGTCAAGACGGAGGTGA-
AAFY01005645  CCCGTCATCTGGGGAGTAGCCAGCCGCCGAA----------------AGGCGGGCTTCCGTGTCAACATACTCGGCTGA--
AAFY01017336  CTTCTCTTCAGGGGAGTAGCTTCCGCGCTTGGTA---------TTCAGGGGCGGACGGT-GGTCAACATACTTGGGGC---
AAFY01027355  CGCTGTCTTAGGGGAGTAGTCTCTCCGGAAGCCT---------GTCTTCCATTGGCGAA-CGTCAACATAATTGATC----

Structure

CH025042      ----------ACATTACCATGGTA-CAAACACCTC---------------------TTGGTTGGCGAGACCATCGATAAGT
CH027617      ----------ACATTACCATGGTA-CAAACACCTC---------------------TTGGTTGGCGAGACCATCGATAAGT
AAFX01006168  -----------AGGGCACCCGGCCGGCTGCAGCA----------------------ATCGGGGCGAGACCACCGCGCGNC
AAFX01013281  GCGGCAACGCCACCCCGCACGGTCCGGTCGGGATGCCGTGGGCCCCGTGGCCCACATGTCAGGGCGAGACCTTCGTTCCGC
AAFX01019015  ----------GGCAGGCCATGGC--GCGCGAGGCAGCCATCGC-----ACGATGGAAGGCCGGGCAAGACCGAAGGCAGGC
AAFX01069316  ---------GACGCGCGCACGGTT-CGGTCGGGTTCCC-----------------GAGGAACAGGCGAGACCTTCGCACGAG
AAFX01111584  GATT--CTTTCCGCGTCCCCGGCCTTATCGACAACCAT--------------GGCGGTTGTAAGCGAGACCTTTGCCTGGC
AAFX01128094  ----------ACCGCCTCCCGGCCTTCCGAATCGGCGC--------------AGTGGCCGAAGGCGAGACCACCACGACAC
AAFY01005645  ------------GAGGCCGTGGTGCGGAAGGAGCGGTCA--------------CCACCGTTCGGGCGAGACCAATGACATTG
AAFY01017336  ---------ACATTCCCCATGGCC-ATCGTGTTTCATTGGTT-------TACCGGTGAAATTAGCAAGACCTGAGGCAGAC
AAFY01027355  ----------CGCAGATCATGGCG-TTCGCAAACCTCAGTCGCCT-GGCGCGACACGGGTTTGACGAGACCTATGACATAC
```

Additional file 15: (A) YYBP/YKOY riboswitch pattern. (B) List of identified YYBP/YKOY riboswitches. (C) Alignment of YYBP/YKOY riboswitch sequences.
