# Supplementary material for: Abundance and functional diversity of riboswitches in microbial communities
Source: BMC Genomics. 2007 Oct 1;8:347. doi: 10.1186/1471-2164-8-347 (PMC2211319; doi:10.1186/1471-2164-8-347)
Supplement: Additional file 16 — Search pattern and sequence alignment of lysine riboswitches. [file 1471-2164-8-347-S16.pdf]

A

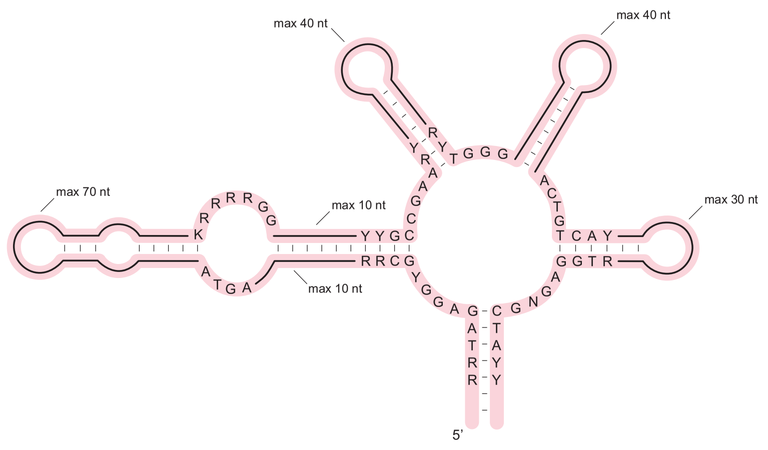

B

Accession	Metagenome	Start position	End position	Regulated function (COG)
CH194310	Sargasso	449	637	COG0527

C

```
Structure
CH194310   TGTCTAGTTAGGAAGAGGTGCATGCACTAGGTAAAGTTTTTGAAGACGCCAACGTCTTTTGATAAAACTTGAGGGAGTAGCATGCCGAAGGTCGTTTTAGAGTTG

Structure
CH194310   GTTCTAAAAGATCTGGTTTCTAAGGCTGAGTCCTTATTAACTGTCATCAAGCTCTGTCTTGATGAAGCGCTTCTAAGGCATTTA
```

Additional file 16: (A) Lysine riboswitch (L-box) pattern. (B) List of identified lysine riboswitches. (C) Alignment of lysine riboswitch sequences.
