# Supplementary material for: Abundance and functional diversity of riboswitches in microbial communities
Source: BMC Genomics. 2007 Oct 1;8:347. doi: 10.1186/1471-2164-8-347 (PMC2211319; doi:10.1186/1471-2164-8-347)
Supplement: Additional file 19 — Search pattern and sequence alignment of YKOK riboswitches. [file 1471-2164-8-347-S19.pdf]

A

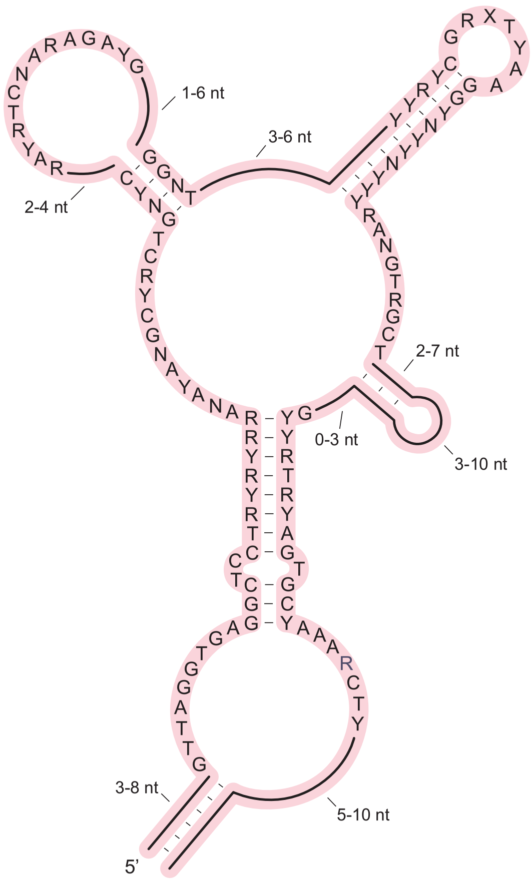

B

Accession	Metagenome	Start position	End position	Regulated function (COG)
AAFX01011285	Soil	692	519	COG2239

C

```
Structure
AAFX01011285   TTCAATAAAGCTTGTTAGGTGAGGTTACTGGTTGAACAGAGGCTGCTGCCCGGAAATGTCGAAAGACACCAATGGGTAGAGCAGGCGTAGTCGGA

Structure
AAFX01011285   TTAAGGCTTCGCATAAGGCAGCTTTCCGGAATCCGGAATAAGTCAACCTGAACTAAAACTCTACGAGGAGCTTCATGCG
```

Additional file 19: (A) YKOK riboswitch pattern. (B) List of identified YKOK riboswitches. (C) Alignment of YKOK riboswitch sequences.
