# Supplementary material for: Abundance and functional diversity of riboswitches in microbial communities
Source: BMC Genomics. 2007 Oct 1;8:347. doi: 10.1186/1471-2164-8-347 (PMC2211319; doi:10.1186/1471-2164-8-347)
Supplement: Additional file 9 — Search pattern and sequence alignment of SAM-riboswitches. [file 1471-2164-8-347-S9.pdf]

A

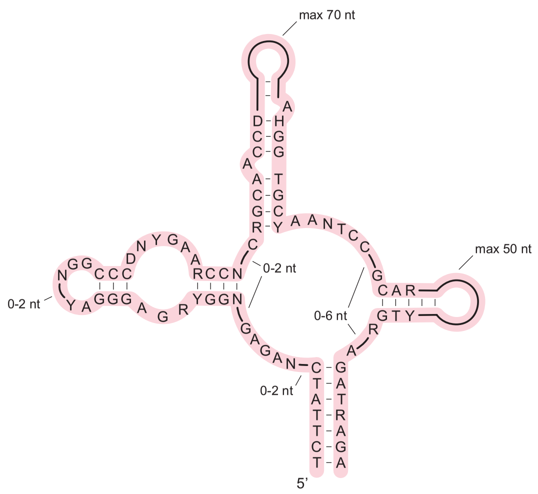

B

Accession	Metagenome	Start position	End position	Regulated function (COG)
CH095766	Sargasso	205	372	COG0646
CH110459	Sargasso	6821	6708	COG0646
CH158823	Sargasso	648	517	COG1897
AAFX01048923	Soil	827	708	COG0460
AAFX01049186	Soil	413	548	COG0498
AAFX01054820	Soil	1103	966	COG0182
AAFX01071693	Soil	991	868	COG0460
AAFX01081827	Soil	78	236	COG0498
AAFY01008477	Whale1	794	652	COG0646
AAFZ01018340	Whale2	185	307	No similar proteins
AAGA01004112	Whale3	792	939	COG2873
AAGA01012172	Whale3	589	469	COG2021

C

```
Structure

CH095766      CTTTTGCCTTGCTTATC-AAGAA-AGACCGAGGGATT-GGGCCCGCTGACGTCT-TAGCAACCTTCTGGTAGGATTTCCGAGCAAAGC
CH110459      TTAGTTTATTACTTATC-AAGAA-AGGTGGAGGGAC--AGGCCCTGTGAAGCCT-TGGCAACCTTT----------------------
CH158823      TAACGTTTATTTGTATC-GAGATGATACTGAGGGATC-TGGCCCTTTGAAGTGTCGAGCAGCCTTTTCA-------------------
AAFX01048923  CGCTGCAGACGCTTATC-CAGAG-TGGCTGAGGGAAC-AGGCCCGTCGAAGCCA-CAGCAACCAGGCGC-------------------
AAFX01049186  ATATCAACGGTCTCATT-CAGAG-TGGCAGAGGGAC--TGGCCCGATGAAGCCA-CGGCAACCGGCCTCGATAAT-------------
AAFX01054820  GGCGGGACGCTCTTATC-GAGAG-TGGTGGAGGGAC--TGGCCCGACGAAGCC--CGGCAACCCGCCGAACCGTC-------------
AAFX01071693  GCGGGCGAGCACTTATCCCAGAG-TGGCTGAGGGAACAGGGCCCGCTGAAGCCA-CAGCAACCAGGCGCGA-----------------
AAFX01081827  ACTGAATACCGCTCATC-CAGAG-GGGTGGAGGGACC--GGCCCTGCGAAGCCC-CGGCAACCACCCGGCTGTGGCGCGTCGCGATCC
AAFY01008477  CGGACTCCGTGCTTATC-AAGAA-AGGTGGAGGGAAT-AGGCCCTGCGAAACCT-TGGCAACCAACTATAAGTCCCGAAT--------
AAFZ01018340  AATTGAGAATCGATATC-GAGAGCGACGTGAGGGTTC-TGGCCCAGTGAACGTC-CGGCAACCTGCCCCA------------------
AAGA01004112  CATCATGCATTCTCATC-AAGAA-AGGTGGAGGGATC-AGGTCCTTTGAAGCCT-TGGCAACCATCTAGTACTTAA------------
AAGA01012172  TTATTGAAACTGTTATA-AAGAA-AGGCAGAGGGATT-AGACCCGATGAAGCCT-TAGCAACCCTTTACTA-----------------

Structure

CH095766      TCGGCAACCTCCCCGGTTTCGGTGCTACCTCCC----ACCAGATGAGCTAATCTCTCTTTCTCTGGTAAGCGATAGTAAAAACCAGAG
CH110459      ----------------GACTGGTGCCAATTCCTGATTTCCAAT-------------------GTGGAATAAGATAAGTTAAAAGAGAT
CH158823      -------------GCATAAAGGTGCTAATGCCAG---CCCAACCGGCAA-------ATGTCGGTGGGAGTCGATGCGACTTGATTGAA
AAFX01048923  -------------AAGCCCAGGTGCTACTTCCT----GCCCGATA--------------AACCGGGA--GAGATAAGAGCCTCGGCAG
AAFX01049186  -------TTCGAGGTGACCAGGTGCCAATTCCA---GCTCGAGCGA-------------AAGTTCGGGAAAAAATGAGAAGAGCAGTTT
AAFX01054820  -------AACGGTTCGGGCGGGTGCCAAGTCCGA--CCTCATTCCGC-----------AGCGGATGGGGAGAGATGAGAGTTCGAAAAG
AAFX01071693  ------------GAGCCCCCGGTGCTACTTCCT----GCCCGATC----------------AACGGGA--GAGATAAGAGCCTCGGTAG
AAFX01081827  TGCGAGGCCGGCCGGGAGATGGTGCCAACTCCG-----GCCTGCGACCAAGG-----TGGCGCAGGG--AAGATGGGGAGAAAGGCCT
AAFY01008477  --GTTCGGGATTGGAAAATCGGTGCTAAATCCTA---TTCGCTTTA---------------AGCGGAA-AAGATAAGTAGAAAAAATAT
AAFZ01018340  ------------TGGGGCAAGGTGCCAATGCCA-----GCCCGGGA-------------AACCGGGA---ACGATGTGGTCCACATGCG
AAGA01004112  ------TTAAGTGCTAGAAAGGTGCTACATCCT---GCTTAGTACTTAAAA--ACTTATGTGCTGAGA-AAGATGAGTGCGAAGAGCT
AAGA01012172  ------------TTAAAGAAGGTGCTAAATTCT----ACCAATT----------------TTTTGGA--TAGATAACAAAAATGAATT
```

Additional file 9: (A) SAM-riboswitch (S-box) pattern. (B) List of identified SAM-riboswitches. (C) Alignment of SAM-riboswitch sequences.
