# Supplementary material for: Abundance and functional diversity of riboswitches in microbial communities
Source: BMC Genomics. 2007 Oct 1;8:347. doi: 10.1186/1471-2164-8-347 (PMC2211319; doi:10.1186/1471-2164-8-347)
Supplement: Additional file 17 — Search pattern and sequence alignment of purine riboswitches. [file 1471-2164-8-347-S17.pdf]

A

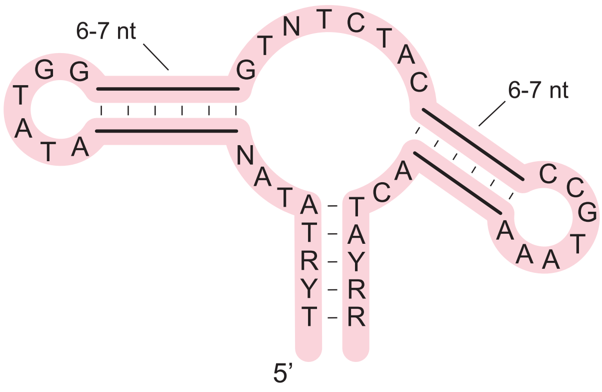

B

Accession	Metagenome	Start position	End position	Regulated function (COG)
AAFX01072182	Soil	8	87	No similar proteins

C

```
Structure
AAFX01072182   GTTGGGGCTCTAATAAAGCCGGTAAAAGGATCNGGCGTTTCTACCAGGCACCGTAAATGTCTGACTATTTGGGGGAAATT
```

Additional file 17: (A) Purine riboswitch (G-box) pattern. (B) List of identified purine riboswitches. (C) Alignment of purine riboswitch sequences.
