# Supplementary material for: Abundance and functional diversity of riboswitches in microbial communities
Source: BMC Genomics. 2007 Oct 1;8:347. doi: 10.1186/1471-2164-8-347 (PMC2211319; doi:10.1186/1471-2164-8-347)
Supplement: Additional file 18 — Search pattern and sequence alignment of GLMS riboswitches. [file 1471-2164-8-347-S18.pdf]

A

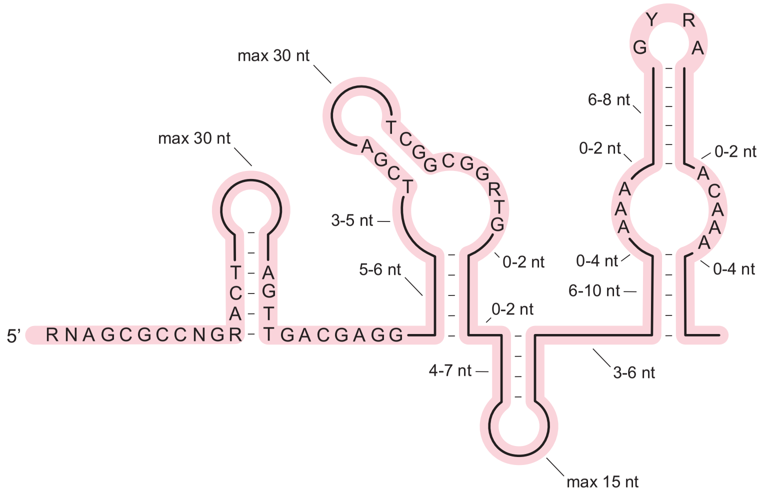

B

Accession	Metagenome	Start position	End position	Regulated function (COG)
AAFX01006262	Soil	175	26	End of DNA fragment
AAFZ01014893	Whale2	396	229	COG0449

C

```
Structure

AAFX01006262   ATAGCGCCAGGACCCGGATCGA--------------GGCTTTCGGGTTGACGAGGTGGGGATTTATCGAATCATTCGGCGGATG
AAFZ01014893   GAAGCGCCAGGACTGGTTGTGATTCGGGTACTATCCCGATGACCAGTTGACGAGGAGAGGGCTTATCGAAGCATTCGGCGGATG

Structure

AAFX01006262   ATCCCCCGGCCGC---ACAGTCGTGATGATGCGCTCAAACCGATTTGGCAACAGATCG-ACAAATGCTCGTCAGCGTGCACATG
AAFZ01014893   GCTCTCCGGCCTGACCGGGGTCGTAACAGCACCGACAAAGTCTGCGGGTGACCGTAGAGACAAAGCGGTGTCGAATCGGTATCT
```

Additional file 18: (A) GLMS riboswitch pattern. (B) List of identified GLMS riboswitches. (C) Alignment of GLMS riboswitch sequences.
